# Automatic and standardized reporting of perioperative MRIs in patients with central nervous system tumors

**DOI:** 10.3389/fneur.2025.1707481

**Published:** 2026-02-06

**Authors:** David Bouget, Mathilde Gajda Faanes, Asgeir Store Jakola, Frederik Barkhof, Hilko Ardon, Lorenzo Bello, Mitchel S. Berger, Shawn L. Hervey-Jumper, Julia Furtner, Albert J. S. Idema, Barbara Kiesel, Georg Widhalm, Rishi Nandoe Tewarie, Emmanuel Mandonnet, Pierre A. Robe, Michiel Wagemakers, Timothy R. Smith, Philip C. De Witt Hamer, Ole Solheim, Ingerid Reinertsen

**Affiliations:** 1 Department of Health Research, SINTEF Digital, Trondheim, Norway; 2 Department of Clinical Neuroscience, University of Gothenburg, Gothenburg, Sweden; 3 Department of Neurosurgery, Sahlgrenska University Hospital, Gothenburg, Sweden; 4 Department of Radiology and Nuclear Medicine, Amsterdam University Medical Centers, Vrije Universiteit, Amsterdam, Netherlands; 5 Institutes of Neurology and Healthcare Engineering, University College London, London, United Kingdom; 6 Department of Neurosurgery, Elisabeth-TweeSteden Hospital, Tilburg, Netherlands; 7 Neurosurgical Oncology Unit, Department of Oncology and Hemato-oncology, Humanitas Research Hospital, Milano, Italy; 8 Department of Neurological Surgery, University of California, San Francisco, San Francisco, CA, United States; 9 Department of Biomedical Imaging and Image-guided Therapy, Medical University Vienna, Wien, Austria; 10 Research Center for Medical Image Analysis and Artificial Intelligence, Faculty of Medicine and Dentistry, Krems, Austria; 11 Department of Neurosurgery, Northwest Clinics, Alkmaar, Netherlands; 12 Department of Neurosurgery, Medical University Vienna, Wien, Austria; 13 Department of Neurosurgery, Haaglanden Medical Center, The Hague, Netherlands; 14 Department of Neurological Surgery, Hôpital Lariboisiére, Paris, France; 15 Department of Neurology and Neurosurgery, University Medical Center Utrecht, Utrecht, Netherlands; 16 Department of Neurosurgery, University Medical Center Groningen, University of Groningen, Groningen, Netherlands; 17 Department of Neurosurgery, Brigham and Women's Hospital, Boston, MA, United States; 18 Harvard Medical School, Boston, MA, United States; 19 Cancer Center Amsterdam, Brain Tumor Center, Amsterdam University Medical Centers, Amsterdam, Netherlands; 20 Department of Neurosurgery, Amsterdam University Medical Centers, Vrije Universiteit, Amsterdam, Netherlands; 21 Department of Neuromedicine and Movement Science, Norwegian University of Science and Technology (NTNU), Trondheim, Norway; 22 Department of Neurosurgery, St. Olavs Hospital, Trondheim University Hospital, Trondheim, Norway; 23 Department of Circulation and Medical Imaging, NTNU, Trondheim, Norway

**Keywords:** 3D segmentation, attention U-net, CNS tumor, RADS, reporting

## Abstract

**Introduction:**

Magnetic resonance (MR) imaging is essential for diagnosing central nervous system (CNS) tumors, guiding surgical planning, treatment decisions, and assessing postoperative outcomes and complications. While recent work has advanced automated tumor segmentation and report generation, most efforts have focused on preoperative data, with limited attention to postoperative imaging analysis.

**Methods:**

This study introduces a comprehensive pipeline for standardized postsurgical reporting in CNS tumors. Using the Attention U-Net architecture, segmentation models were trained, independently targeting the preoperative tumor core, non-enhancing tumor core, postoperative contrast-enhancing residual tumor, and resection cavity. In the process, the influence of varying MR sequence combinations was assessed. Additionally, MR sequence classification and tumor type identification for contrast-enhancing lesions were explored using the DenseNet architecture. The models were integrated seamlessly into an automated and standardized reporting pipeline, following the RANO 2.0 guidelines. Training was conducted on multicentric datasets comprising 2000 to 7000 patients, incorporating both private and public data, using a 5-fold cross-validation.

**Results:**

Evaluation included patient-, voxel-, and object-wise metrics, with benchmarking against the latest BraTS challenge results. The segmentation models achieved average voxel-wise Dice scores of 87%, 66%, 70%, and 77% for the tumor core, non-enhancing tumor core, contrast-enhancing residual tumor, and resection cavity, respectively. Classification models reached 99.5% balanced accuracy in MR sequence classification and 80% in tumor type classification.

**Discussion:**

The pipeline presented in this study enables robust, automated segmentation, MR sequence classification, and standardized report generation aligned with RANO 2.0 guidelines, enhancing postoperative evaluation and clinical decision-making. The proposed models and methods were integrated into Raidionics, open-source software platform for CNS tumor analysis, now including a dedicated module for postsurgical analysis.

## 1 Introduction

Brain tumors encompass a diverse range of neoplasms with highly variable prognoses, ranging from benign to highly aggressive forms. The World Health Organization (WHO) currently classifies over 100 subtypes based on molecular and histological profiles (1). While some patients experience prolonged survival, many face rapid neurological and cognitive decline (2). Accurate tumor characterization is crucial for prognosis, treatment planning, and surgical decision-making. However, the inherent biological complexity of CNS tumors presents significant challenges. CNS tumors are broadly categorized as primary or secondary. Primary tumors, such as gliomas and meningiomas, originate within the brain or its supporting tissues. Gliomas include the most aggressive and treatment-resistant forms (e.g., glioblastoma), as well as slowly progressive yet highly infiltrative forms ultimately undergoing malignant transformation (e.g., diffuse lower-grade) (3, 4). Secondary tumors result from metastatic spread to the brain. Magnetic Resonance (MR) imaging is essential for tumor diagnosis, prognosis estimation, and treatment planning. Imaging-derived features such as volume, location, and structural involvement guide surgical resection, estimate postoperative risks, and inform adjuvant therapy (5). These features also underpin predictive models for clinical research and personalized care (6–8). Despite advances in imaging, tumor characterization remains largely subjective. Manual segmentation, the current gold standard, remains labor-intensive and prone to intra- and inter-rater variability (9) and thus rarely performed in routine practice. Instead, tumor attributes are often estimated visually or using crude methods (e.g., eyeballing or short-axis diameter measurements), introducing significant inconsistencies and reducing clinical utility (10). The lack of automated and standardized segmentation limits the integration of imaging-based biomarkers into clinical workflows, and represents a weakness for clinical trial assessment both at baseline and for estimating treatment responses. Robust computational methods are needed to improve precision, reproducibility, and efficiency, bridging the gap between imaging, molecular diagnostics, and personalized treatment strategies.

Post-operative MRI is critical for evaluating surgical outcomes, planning adjuvant therapy, and monitoring disease progression. However, altered anatomy, resection cavities, and postoperative blood products greatly complicate segmentation. Residual tumors are often small and very fragmented compared to preoperative tumor cores. The BraTS challenge, long focused on preoperative segmentation, was extended in 2024 to postoperative cases (11), introducing annotations for the enhancing tissue (ET), non-enhancing tumor core (NETC), surrounding non-enhancing FLAIR hyperintensity (SNFH), and resection cavity (RC). Top-performing methods employed CNN and Transformer-based architectures [e.g., nnU-Net (12), Swin U-Netr (13)], ensembles [e.g., STAPLE (14)], and synthetic data augmentation (15). Nonetheless, performance remains significantly lower than in the preoperative setting, with lesion-wise Dice scores reaching up to 78% for NETC, 76% for ET, and 71% for RC. Other studies focusing on residual tumor segmentation attempted training from scratch (16), through active learning (17–19), or transfer learning from a preoperative model (20). In those studies, leveraging local datasets not publicly available, lower Dice scores were reported, at around 50%–60% Dice for ET. This highlights a considerable performance gap compared to the typical BraTS challenge results, underscoring greater image and annotation variability in the postoperative setting. Postprocessing is often applied to remove artifacts and enforce anatomical consistency (e.g., NETC within TC). Resection cavity segmentation remains underexplored: studies on limited datasets reported 80% Dice score using a DenseNet variation (21) or 3D U-Net with longitudinal data (22). Alternatively, resection cavities were simulated before being segmented using self-supervised and semi-supervised learning (23). Overall, postoperative segmentation tasks remain unsolved, highlighting the need for more robust methods. Beyond segmentation, standardized imaging reports are essential. The lack of structured reporting systems for glioma patients was identified as a major limitation in 2018 (24). Although structured assessment has the potential to improve patient care and enhance both communication and decision-making, few solutions have been proposed. A preoperative reporting framework was introduced in the Raidionics platform (25), leveraging features from automatic segmentation for major CNS tumor types. Regarding postoperative assessment, prior works proposed new scoring methods and assessment (24, 26), but these were not derived from automated MR analysis. With growing interest in postoperative segmentation and emerging models for ET and RC, there is a clear opportunity for advancing postoperative standardized and automatic reporting into readily available solution.

This work introduces a comprehensive pipeline for automated postsurgical assessment in CNS tumors, with the following contributions: (i) development of robust segmentation models for tumor core, non-enhancing tumor core, residual tumor, and resection cavity, (ii) thorough validation and benchmarking against BraTS, including ablation studies on required MR sequences, (iii) extension and refinement of image-based standardized reporting in line with RANO 2.0 guidelines, and (iv) integration into the Raidionics software, providing access to the latest segmentation, classification, and reporting methods.

## 2 Data

**Figure 1:**
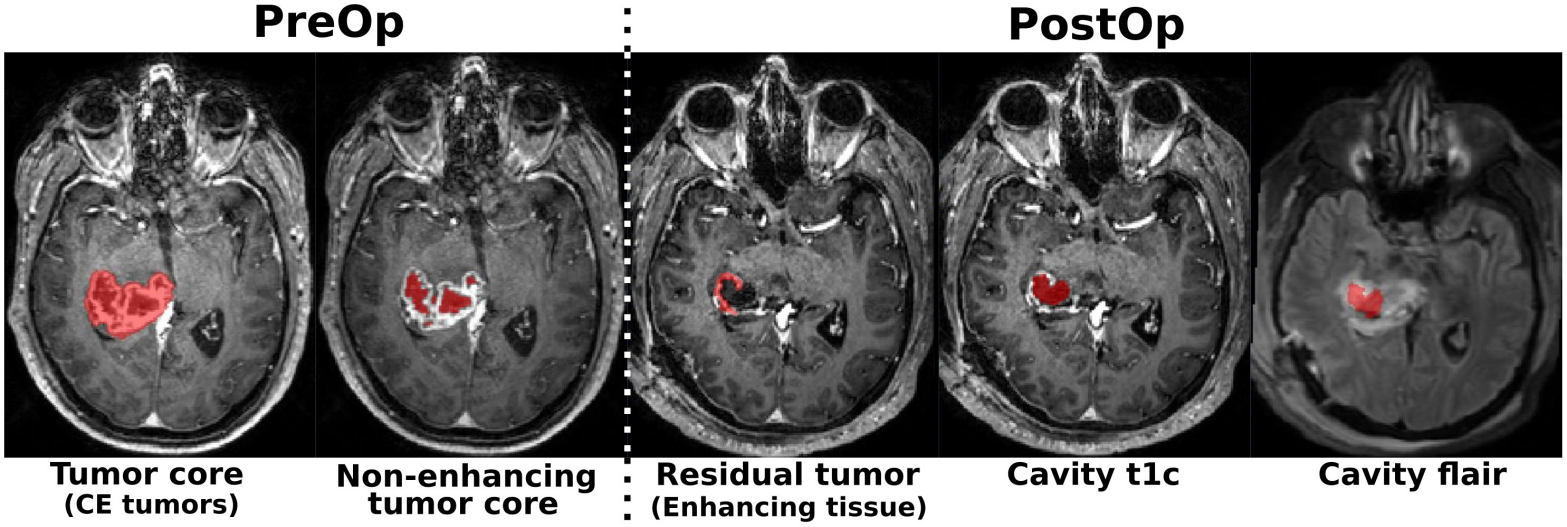
Illustration of the different structures targeted for model segmentation training: tumor core (for contrast-enhancing tumors), non-enhancing tumor core, postoperative residual tumor (enhancing tissue), resection cavity in t1c MR scan, and resection cavity in FLAIR MR scans **(from left to right)**.

All data used in this study were obtained from a previously described private dataset (27), and the publicly available datasets from the BraTS challenges (11, 28–30). Given the diverse segmentation and classification tasks addressed in this study, different specific subsets were compiled, as detailed below. An overview of the segmentation subsets is provided in Table 1. First, for the MR sequence classification task, a total of 1, 000 MR scans were randomly selected for each combination of MR sequence type and acquisition timestamp (i.e., preoperatively and postoperatively), resulting in a final subset of 8, 000 MR scans. The MR sequence types considered are: gadolinum-enhanced T1-weighted (noted t1c), T1-weighted (noted t1w), FLAIR (noted flair), and T2-weighted (noted t2w). Second, for the contrast-enhancing tumor type classification, a total of 500 pre-operative t1c MR scans were randomly selected for each class (i.e., glioblastoma, meningioma, and metastasis), resulting in a final subset of 1, 500 MR scans. Finally, for the segmentation task, four distinct subsets were assembled using a mixture of patient data at different timepoints. Each subset targets a specific structure to segment, the tumor core for contrast-enhancing tumors (i.e., TC) in dataset A, the non-enhancing tumor core (i.e., NETC) in dataset B, the residual tumor (or enhancing tissue) in dataset C, and finally the resection cavity in dataset D (as illustrated in Figure 1). In Table 1, the total number of unique patients, as well as the total number of investigation from different timestamps, are summarized. Additionally, the incremental decrease of number of investigations, arising from the inclusion of additional MR sequences, is also reported. Different cut-off values were applied to determine whether the segmentation target was sufficiently visible in a given MR scan to be considered a positive sample: 0.175 ml for postoperative residual tumor (31), 0.05 ml for NETC, 0.1 ml for resection cavity, and 0.1 ml for TC. For the private data, tumor core, residual tumor, and resection cavities were manually segmented in 3D by trained raters. On preoperative t1c scans, the tumor core region was defined as gadolinum-enhancing tissue, necrosis, and cysts. On postoperative t1c scans, the enhancing tissue region was defined as gadolinum-enhancing tissue. Finally, the resection cavity was defined as regions with a signal isointense to cerebrospinal fluid, potentially including air, blood, or proteinaceous materials when recent. A more detailed description of each cohort and dataset is available in Supplementary Section 1.

**Table 1:** Overview of the datasets used for the different segmentation tasks.

**Name**	**Target**	**Timestamps**	**Patients**	**Sources**	**Positives**	**t1c**	**+t1w**	**+flair**	**+t2w**	**Volume (ml)**
A	Tumor core	7, 171	7, 171	17	7, 106	7, 171	-	-	-	30.28 ± 27.95
B	NETC	4, 427	3, 724	1^+^	2, 507	4, 427	4, 427	4, 427	4, 427	05.43 ± 13.83
C	Residual tumor	2, 648	1, 945	16	1, 674	2, 648	2, 616	2, 463	2, 224	04.98 ± 06.77
D	Resection cavity	2, 275	1, 572	16	2, 048	1, 969	1, 926	1, 859 (306^*^)	1, 826	15.95 ± 19.69

A - Pre-operative tumor core (for contrast-enhancing CNS tumor), B - Pre/post-operative non-enhancing tumor core (NETC), C - Post-operative residual tumor, D - Resection cavity. ^*^For 306 lower-grade glioma patients, the resection cavity is only available in FLAIR MR scans. (+) The BraTS challenge data is considered as a single data source even if collected from multiple hospitals.

## 3 Methods

### 3.1 Segmentation models training

**Figure 2:**
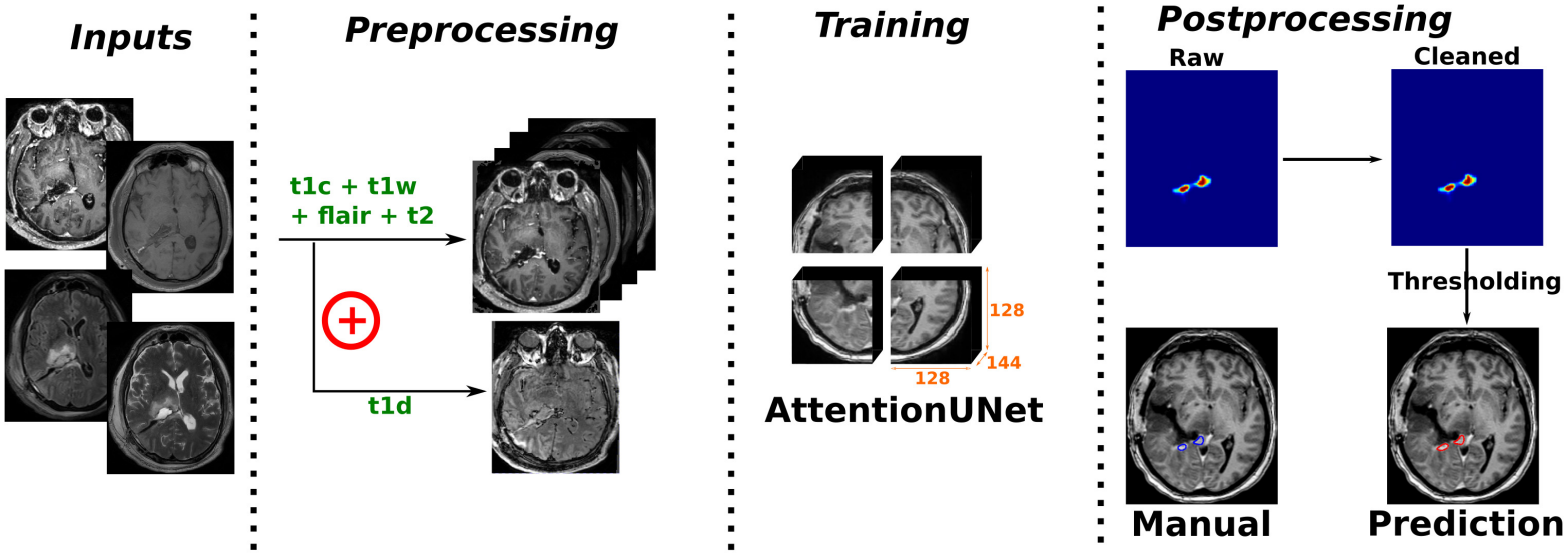
Illustration of the segmentation model training pipeline including the following four steps: MR sequence input selection, preprocessing, training using the Attention U-Net architecture, and finally post-processing.

Segmentation models were trained following the pipeline illustrated in Figure 2, with preprocessing variations based on the available input MR sequences. Each step of the pipeline is further detailed in the following paragraphs.

#### 3.1.1 Input selection and preprocessing

Given the possibility for one or multiple MR scans to be available for any given patient, incremental combinations of input MR sequences are made possible. Additionally, pairs of input sequences can be subtracted to generate new input channels. The inclusion of such subtraction-based inputs is indicated by an additional marker *(d)* within the experiment name (e.g., *t1d* for subtraction between t1c and t1w scans). The subtraction between t1c and t1w scans is inspired by the best-performing team for meningioma segmentation in the BraTS 2024 challenge and by the official annotation protocol from the BraTS challenge (11). Such input was provided to help identify subtle areas of enhancement and to distinguish areas of intrinsic T1 hyperintensity from enhancement.

The following preprocessing steps were applied in the specified order: (i) resampling to an isotropic voxel spacing of 1mm^3^ using first-order spline interpolation, (ii) tight cropping around the patient's head to exclude the background, (iii) subtraction of two input sequences to create a new difference input channel (when applicable), (iv) intensity clipping within the range [0, 99.5]%, and (v) zero-mean normalization of all nonzero values.

#### 3.1.2 Architecture design and training strategy

The Attention U-Net architecture (32) has been used in this work with the following specifications: 5 levels, filter sizes of [16, 64, 128, 256, 512], instance normalization, a dropout rate of 0.2, and an input size of 128 × 128 × 144 voxels.

The loss function was the combination of Dice and Cross-Entropy, with a sigmoid final activation for single-class segmentation tasks (i.e., including the background). Model training was conducted using the AdamW optimizer with an initial learning rate of 5*e* − 4, combined with an annealing scheduler. Training was performed over 800 epochs with a batch size of 16 elements, using a 2 step gradient accumulation strategy to achieve an effective batch size of 32 elements.

For data augmentation, a random crop of 128 × 128 × 144 voxels was applied to each input sample. Subsequently, a random combination of geometric, each with a 50% probability to happen over any given axis, and intensity-based transformations with a 50% probability were performed. The geometric transformations include flipping, rotation within the range [−20°, 20°], translation of up to 20% of the axis dimension, and zoom scaling of up to 15%. The intensity-based transformations include intensity scaling and shifting (up to 10%), gamma contrast adjustments in the range [0.5, 2.0], Gaussian noise addition, and patch dropout or inversion with patch sizes of 10 × 10 × 10 voxels and up to 75 elements.

#### 3.1.3 Inference and postprocessing

For the inference step, a sliding-window approach with 50% overlap between consecutive patches along each spatial dimension was performed. Unlike the patch size employed during training, the inference patch size was set to 160 × 160 × 160 voxels. During the inference process, no data augmentation was performed over the input samples. Subsequently, a two-step postprocessing refinement was designed to clean the predictions. With the first step, only the prediction probabilities lying inside a binary mask of the brain location were kept. Second, noise in prediction probabilities was removed by identifying connected component predictions with an area lower than 0.05*ml*^3^ or not visible in consecutive 2D slices.

### 3.2 Single timepoint and surgical standardized reporting

**Figure 3:**
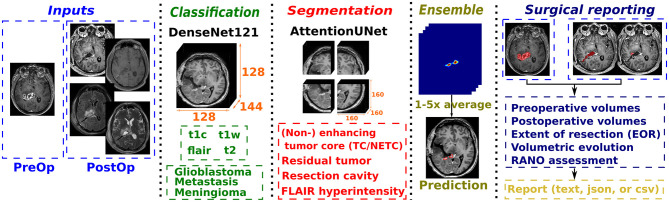
Pipeline illustration for surgical reporting using any given set of input MR scans. The different steps are: MR sequence classification, contrast-enhancing tumor type classification, input-agnostic structures segmentation, model ensembling, and finally reporting generation (per timepoint and post-surgical).

The proposed pipeline for standardized surgical reporting, illustrated in Figure 3, starts with a classification step to automatically identify the MR sequence for all provided input scans and the type of contrast-enhancing tumor (if applicable). Then, segmentation of multiple structures is performed using the latest models, with the possibility to include an extra step of ensembling to generate more robust results. Finally, a surgical reporting can be generated, complementing the standardized reporting computed for each timepoint. Each step is described in-depth in the remainder of the section.

#### 3.2.1 MR scan sequence and contrast-enhancing tumor type classification

In order to automatically assign the proper MR sequence to each input scan and identify the contrast-enhancing tumor type, 3D classification model training was performed. The DenseNet121 architecture (33) has been used with 64 filters in the first convolution layer, a growth rate of 32, batch normalization, and an input size of 128 × 128 × 144 voxels. A single input was provided to the DenseNet121 model, the original t1c MR scan.

The loss function was Cross-Entropy and the multi-class accuracy was employed as metric. Model training was conducted using the AdamW optimizer with an initial learning rate of 5*e* − 4, combined with an annealing scheduler. Training was performed over 800 epochs with a batch size of 8 elements, using a 4 step gradient accumulation strategy to achieve an effective batch size of 32 elements. The same data augmentation techniques as described in the previous section were used.

#### 3.2.2 Segmentation and model ensembling

The inference process is the one previously described and by default no runtime data augmentation nor model ensembling is performed. Both options can be enabled by the user in order to improve the robustness of the segmentation results only at the expense of longer processing time. The available runtime data augmentation techniques, using the same parameters as during training, include axis flipping, rotation, and gamma contrast. For model ensembling, the segmentation results from one to five models can be combined by returning either the mean probability (i.e., *average* option) or the maximum probability (i.e., *amax* option) for each voxel.

#### 3.2.3 Global structures' segmentation refinement

As each structure segmentation model was trained independently, a refinement step leveraging global context is appropriate to ensure consistency across all structures. In addition to the models described in this study, the FLAIR hyperintensity (i.e., SNFH) segmentation models, trained using the training protocols (34), were included in this step.

For contrast-enhancing tumors: Preoperatively, the tumor core mask is kept unchanged and used as reference. The NETC mask is refined by retaining only regions that overlap with the tumor core mask, and the SNFH mask is modified by subtracting the tumor core region. The non-overlapping tumor core and SNFH masks can be combined to form the whole tumor mask. Postoperatively, the residual tumor mask is kept unchanged and used as reference. The resection cavity and enhancing tissue masks are subtracted from the SNFH mask.

For non contrast-enhancing tumors: No preoperative tumor core or postoperative residual tumor masks are available. In both preoperative and postoperative settings, the SNFH mask serves as the whole tumor mask. Specifically postoperatively, the resection cavity mask is subtracted from the SNFH mask.

#### 3.2.4 Standardized reporting

Standardized reports for single timepoints (i.e., preoperative and postoperative) are computed in the same way as described in our previous study (27). The major variation comes from the selection of segmentation models used for the different use-cases, when only a single tumor model was available before. For contrast-enhancing tumors, the tumor core segmentation model operating over gadolinum-enhanced T1-weighted MR scans is used preoperatively and the residual tumor segmentation model is used postoperatively over up to four MR sequences. For non contrast-enhancing tumors, the unified SNFH segmentation model is used (34). For all tumor types, the unified resection cavity and necrosis segmentation models are used over up to four MR sequences. The set of computed features has been extended to include diameter characteristics (i.e., long-axis, short-axis, Feret, and equivalent area), tumor-to-brain ratio, and the Brain-Grid classification system for cerebral gliomas (35).

The standardized surgical report features the same distinction between contrast-enhancing and non contrast-enhancing tumors. Preoperative and postoperative volumes for all segmented structures are reported, when applicable. In addition, post-surgical volumetric evolution percentages are reported for each segmented structure, which is the equivalent to the extent-of-resection when the considered structure is the tumor. Finally, the overall surgical assessment, i.e., complete, near total, or subtotal resection, is provided to complement the standardized surgical report, following the latest RANO guidelines (31).

## 4 Validation studies

In this work, the focus lies primarily on assessing segmentation and classification models' performance, and as such four experiments were conducted. A single training protocol has been followed for all presented models, namely a 5-fold cross-validation. All datasets were randomly split into 5 folds, simply enforcing for a single patient's data not to be featured in multiple folds when MR scans were available at multiple timestamps. During training, 3 folds were used as training set, one fold as validation set, and the remaining fold as test set, following an iterative process. For the validation studies, both postprocessing steps were used for the residual tumor structure, and only the first step was applied for all other structures.

### 4.1 Metrics

For quantifying and comparing models' performances, the following patient-wise, voxel-wise, and object-wise metrics were computed. Each metric was computed between the ground truth volume and a binary representation of the probability map generated by a trained model. The binary representation is computed for ten different equally-spaced probability thresholds in the range [0, 1]. For the probability threshold providing the best results, pooled estimates computed from each fold's results are then reported for each metric (36) to provide overall results, reported as mean and standard deviation (indicated by ±) in the tables. Voxel-wise and object-wise results are only reported for the positive samples following the definition provided below.

#### 4.1.1 Patient-wise

Patient-wise metrics assess the classification ability of a given segmentation or classification model. For segmentation models, the cut-off volume values presented in the Data section were used to determine positive cases (i.e., including the structure of interest) from negative cases (i.e., not include the structure of interest). Furthermore, a voxel-wise Dice overlap of simply 0.1% between model prediction and ground truth was required for a positive case to be considered true positive (TP). From the identification of true/false positive/negative samples at a patient level, the following metrics were then computed: recall, precision, specificity, and balanced accuracy (noted bAcc).

#### 4.1.2 Voxel-wise

Voxel-wise metrics assess the ability of a segmentation model by considering each voxel independently. The following metrics were computed between the ground truth volume and the binary model prediction: Dice score, recall, precision, and 95th percentile Hausdorff distance (noted HD95).

#### 4.1.3 Object-wise

Object-wise metrics assess the ability of a segmentation model to detect all components of the structure to segment (i.e., multiple tumor components). The trained models not being instance segmentation models, a connected components approach coupled to a pairing strategy was employed to associate ground truth and model predictions' components. A strict assignment was performed using the Hungarian algorithm. A minimum component size of 75 voxels (down to 50 voxels for the NETC structure) was enforced and objects below the threshold were discarded. Dice score, recall, precision, and 95th percentile Hausdorff distance were computed.

### 4.2 Experiments

First, (i) classification performance analyses for MR sequence and tumor type identification were executed. Next, (ii) a performance analysis of the contrast-enhancing tumor core segmentation model and NETC segmentation model was performed. Then, (iii) a postoperative segmentation performance experiment was conducted to identify the impact of using a varying number of MR sequences as input and present the best performing models for the enhancing tissue and resection cavity categories. Ensuing, (iv) a detailed analysis was carried out to highlight performance differences across the different cohorts in our dataset, as well as in comparison with the BraTS challenge for external benchmarking. Finally, (v) a comprehensive investigation of generalizability will be performed with identification of hard cases and annotation inconsistencies. Sensitivity analysis will also be conducted to evaluate the impact of detection criteria.

## 5 Results

All experiments were performed using computers with the following specifications: Intel Core Processor (Broadwell, no TSX, IBRS) CPU with 16 cores, 64GB of RAM, Tesla A40 (46 GB), or A100 (80GB) dedicated GPUs, and NVMe hard-drives. Training and inference processes were implemented in Python 3.11 using PyTorch v2.4.1, PyTorch Lightning v2.4.0, and MONAI v1.4.0 (37). The source code used for computing the metrics and performing the validation studies, all trained models, and inference code, are publicly available through our Raidionics platform (25).

### 5.1 Classification performances

Classification performances for both MR sequence and tumor type tasks are summarized in Table 2. From the use of a large dataset of 8, 000 samples, MR sequence classification performances are almost perfect with a 99% balanced accuracy score. On the other hand, classification performances for the contrast-enhancing tumor type are only reaching 85% balanced accuracy. The results can be explained by the use of a relatively smaller dataset, when compared to the MR sequence classification dataset, and by the more difficult nature of the task when only leveraging the t1c MR scans.

**Table 2:** Multiclass-averaged classification performance for distinguishing between MR sequence type (Sequence) and between contrast-enhancing tumor type (Type).

**Task**	**Recall**	**Precision**	**Specificity**	**F1-score**	**Accuracy**	**bACC**
Sequence	98.83 ± 0.19	98.84 ± 0.19	99.61 ± 0.06	98.83 ± 0.19	99.42 ± 0.09	99.22 ± 0.13
Type	80.06 ± 2.27	80.46 ± 2.80	89.99 ± 1.23	80.02 ± 2.42	86.67 ± 1.61	85.02 ± 1.75

**Figure 4:**
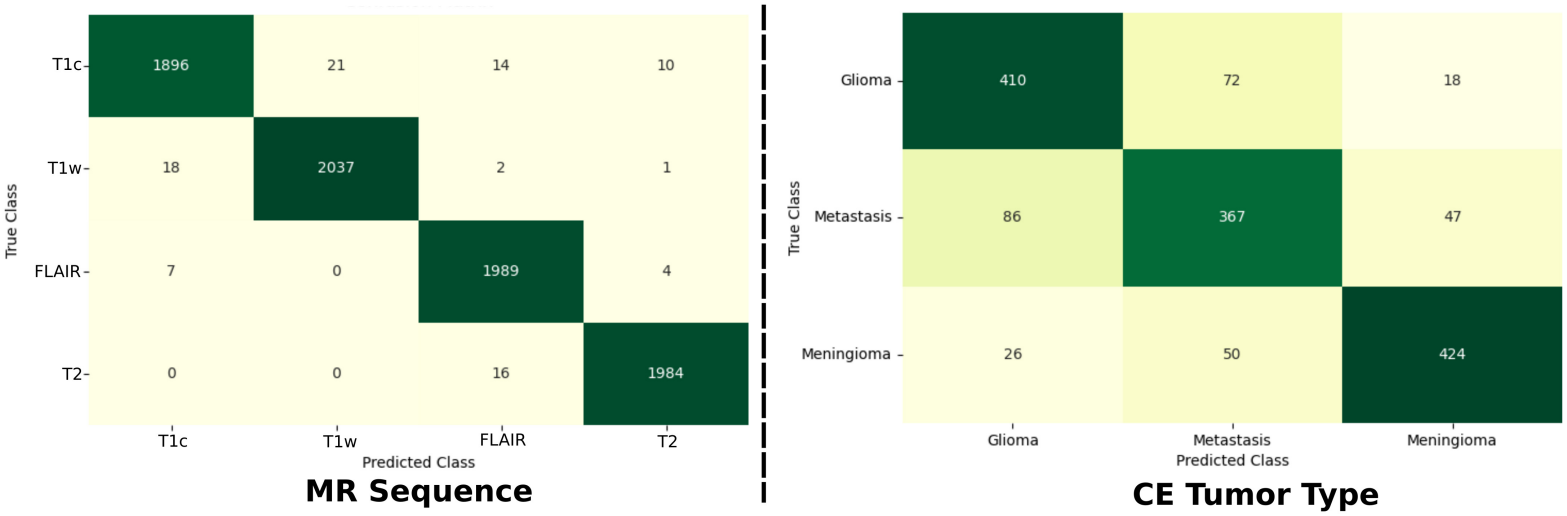
Confusion matrices for both classification tasks where the predicted class is shown in the x-axis and the ground truth class in the y-axis.

**Figure 5:**
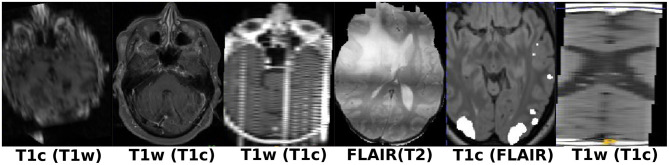
Examples of misclassified MR scans due to extreme motion artifacts, reconstruction artifacts, noise, or cropping. The predicted MR sequence is indicated first and the ground truth MR sequence is specified in parenthesis.

To further investigate where the classification models failed, confusion matrices were computed for each task (cf. Figure 4). For the MR sequence task, frequent misclassifications occurred between gadolinum-enhanced T1-weighted and regular T1-weighted MR scans. Similarly, confusion was common between FLAIR and T2 MR sequences. These results are expected, as each of these pairs shares similar physical imaging characteristics. Upon detailed review of the 93 misclassified cases, it was found that in 61 instances the ground truth labels were incorrect, while the model predictions were actually accurate. Therefore, only 32 cases (i.e., 0.4%) were truly misclassified. These errors were primarily attributed to extreme cropping, imaging artifacts, noise, or blurriness (as illustrated in Figure 5). Regarding the tumor type task, both glioblastomas and meningiomas were usually correctly classified. The largest confusion came from the metastasis group, more often misclassified as either of the two other groups.

### 5.2 Contrast-enhancing tumor core and NETC segmentation performances

Tumor core segmentation performance in preoperative MR scans for contrast-enhancing tumors are reported in Table 3. Overall patient-wise metrics indicate an almost perfect detection rate with 99% recall and precision. However, from the heavy imbalance between positive and negative samples in dataset A, the balanced accuracy and specificity were negatively impacted. Both voxel-wise and object-wise Dice scores reached high values above 85%, indicating strong efficiency both on average and for each tumor type individually. The different metrics are quite homogeneous across the board. The lowest voxel-wise Dice score (85%) was obtained for the metastasis group while the highest (89%) was obtained for the glioma group. Supported by the average volumes reported in Supplementary Table S1, larger tumors exhibit higher Dice scores on average. Interestingly, the object-wise Dice score for the metastasis group is almost as high as for the other two groups. Despite metastasis being on average smaller and more fragmented than meningioma and glioma, the results showcase high and stable segmentation performance. A deeper performance investigation based on tumor type and cohort is available in Supplementary Table S5 and Supplementary Figure S1.

**Table 3:** Preoperative contrast-enhancing tumor core segmentation performances, over t1c MR scans from dataset A.

**Type**	**# Samples**	**Patient-wise**				**Voxel-wise**				**Object-wise**			
		**Recall**	**Precision**	**Specificity**	**bAcc**	**Dice**	**Recall**	**Precision**	**HD95**	**Dice**	**Recall**	**Precision**	**HD95**
GLI	3, 655	99.67 ± 00.22	99.81 ± 00.11	26.89 ± 38.87	63.28 ± 19.38	89.01 ± 14.02	89.75 ± 13.82	90.36 ± 13.77	4.29 ± 10.19	88.27 ± 14.11	88.93 ± 14.42	90.25 ± 13.29	2.88 ± 3.75
MEN	2, 278	98.29 ± 00.65	99.91 ± 00.17	79.02 ± 40.00	88.65 ± 19.89	85.94 ± 20.67	85.61 ± 21.84	88.41 ± 19.64	7.93 ± 20.55	86.32 ± 20.35	85.74 ± 21.74	91.16 ± 14.49	2.42 ± 3.92
MET	1, 214	98.83 ± 00.76	99.16 ± 00.44	59.88 ± 22.42	79.36 ± 10.99	85.05 ± 15.41	83.93 ± 16.71	88.92 ± 14.11	10.36 ± 21.72	83.67 ± 20.82	86.29 ± 14.91	90.41 ± 13.24	1.49 ± 1.30
All	7, 171	99.09 ± 00.08	99.73 ± 00.07	39.46 ± 16.84	69.27 ± 08.39	87.32 ± 16.80	87.44 ± 17.43	89.44 ± 16.06	6.49 ± 16.55	86.84 ± 17.67	87.46 ± 17.27	90.52 ± 13.77	2.52 ± 3.66

The tumor types are glioma (GLI), meningioma (MEN), and metastasis (MET).

Segmentation performances for the NETC category are reported in Table 4. The incremental addition of MR scans as input seemed to have a marginal effect only on the average performances. Both average pixel-wise and object-wise Dice scores went from 65.6% with one input sequence to 66.8% with three. Given the higher negative-to-positive sample ratio in dataset B, patient-wise specificity and balanced accuracy are above 70% and 80% respectively. The final addition of FLAIR slightly worsened overall performance with Dice score values around 65.5% yet providing a higher recall than using t1c sequences only. The very fragmented nature of the NETC structure is clearly visible from the large difference between pixel-wise and object-wise HD95 values. By removing all fragments below a predefined volume threshold, as conventionally done in the BraTS challenge, the distance between the largest paired components is drastically reduced. A deeper performance investigation based on tumor type and NETC volume is available in Supplementary Tables S6, S7, Supplementary Figures S2, S6.

**Table 4:** Non-enhancing tumor core segmentation performances over dataset B, for different sets of MR sequences as input.

**Inputs**	**Patient-wise**				**Voxel-wise**				**Object-wise**			
	**Recall**	**Precision**	**Specificity**	**bAcc**	**Dice**	**Recall**	**Precision**	**HD95**	**Dice**	**Recall**	**Precision**	**HD95**
t1c	94.24 ± 01.47	82.20 ± 01.15	73.35 ± 01.58	83.80 ± 00.92	65.64 ± 30.84	70.78 ± 33.30	69.45 ± 29.30	10.21 ± 16.88	65.61 ± 31.89	75.03 ± 32.69	75.35 ± 25.96	3.75 ± 4.70
+ t1wt1d	94.99 ± 01.19	80.86 ± 01.38	70.65 ± 01.71	82.82 ± 01.18	66.11 ± 30.47	71.99 ± 32.55	68.90 ± 29.05	10.37 ± 17.39	66.27 ± 31.23	76.71 ± 31.62	74.21 ± 26.40	3.78 ± 4.74
+ flair	95.08 ± 01.53	81.21 ± 03.26	71.17 ± 05.16	83.13 ± 01.88	66.72 ± 30.02	72.15 ± 32.28	69.93 ± 28.59	10.47 ± 17.99	66.85 ± 31.22	76.69 ± 31.52	75.03 ± 25.99	3.71 ± 4.89
+ t2w	95.97 ± 01.37	77.57 ± 02.12	63.76 ± 03.10	79.87 ± 00.93	65.30 ± 29.94	75.22 ± 31.55	64.71 ± 29.13	10.78 ± 18.62	65.43 ± 30.56	79.35 ± 30.78	68.94 ± 27.70	3.61 ± 4.37

### 5.3 Postoperative segmentation performances

First, segmentation performances for the contrast-enhancing residual tumor category, in postoperative MR scans, are reported in Table 5. The best performances are achieved for the model trained using all four input MR sequences with a 70% pixel-wise and object-wise average Dice score. An overall decrease of 7% Dice can be noticed when training a model using a single MR sequence as input, and each input sequence iteratively boosts performances by 1%–2% Dice. Looking at the predictive performances, the model using only t1c inputs exhibits oversegmentation and struggles to properly identify patients with residual tumor from patients without. A substantial improvement in patient-wise specificity and balanced accuracy is obtained from using both t1c and t1w MR sequences as input with a respective 13% and 6.5% increase. Having access to information from two different but highly correlated MR sequences helped the model to better differentiate between residual tumor and blood products. However, the best patient-wise specificity score reached only 70% with all four input MR sequences, highlighting the struggle not to oversegment given the very fragmented nature of residual tumor.

**Table 5:** Overall segmentation performance summary for contrast-enhancing residual tumor over dataset C, with incremental inclusion of MR sequences as input.

**Inputs**	**Patient-wise**				**Voxel-wise**				**Object-wise**			
	**Recall**	**Precision**	**Specificity**	**bAcc**	**Dice**	**Recall**	**Precision**	**HD95**	**Dice**	**Recall**	**Precision**	**HD95**
t1c	97.18 ± 01.12	77.01 ± 01.03	50.70 ± 03.46	73.94 ± 01.28	63.26 ± 26.77	70.14 ± 27.26	64.30 ± 29.11	12.85 ± 21.27	64.27 ± 26.42	71.25 ± 27.99	68.77 ± 27.49	5.52 ± 6.18
+ t1wt1d	95.31 ± 01.08	81.37 ± 04.29	63.60 ± 06.61	79.46 ± 03.01	66.10 ± 26.53	70.58 ± 27.80	68.61 ± 28.10	11.14 ± 17.31	67.06 ± 26.40	72.57 ± 27.76	72.99 ± 25.82	4.71 ± 5.27
+ flair	95.17 ± 01.32	83.27 ± 03.18	68.06 ± 07.85	81.61 ± 03.46	67.72 ± 26.36	70.85 ± 27.65	70.86 ± 27.70	10.37 ± 17.74	68.10 ± 26.55	72.35 ± 27.89	74.91 ± 25.16	4.52 ± 5.51
+ t2w	95.27 ± 00.75	84.03 ± 02.51	70.61 ± 04.78	82.94 ± 02.40	70.24 ± 25.97	73.49 ± 26.60	73.06 ± 27.18	8.66 ± 16.05	70.42 ± 26.02	74.93 ± 26.71	77.17 ± 23.81	4.32 ± 5.38

Segmentation performances for the resection cavity in postoperative MR scans are reported in Table 6. The resection cavity often comprise a single region and as such object-wise metrics are closely matching the pixel-wise metrics. The lowest object-wise Dice score is obtained when using a single FLAIR MR scan as input, reaching only 66.5%. The peculiarity of this use-case is the mixture of resection cavities in patients suffering from glioblastoma or diffuse lower-grade glioma, potentially rendering the task more difficult from higher brain structure and appearance variability. Regarding the sequential inclusion of MR sequence from t1w to t2w, performances are almost identical using a single input or the four MR sequences, hovering around 78% object-wise Dice. The specific addition of the t1d input did not provide any difference in voxel-wise or object-wise performances. A slight improvement in patient-wise metrics can be noticed over specificity and accuracy. From the patient-wise results, the classification ability is relatively poor with around 25% specificity and 60% balanced accuracy. As highlighted by the dataset distribution in Supplementary Table S4, the positive rate lies at 90%. Hence, very few negative samples are provided to the model during training for mitigating such oversegmentation behavior. Yet, the patient-wise precision is relatively high with 90%, indicating that not many patients were misclassified regarding the presence or absence of a resection cavity. The specificity values are negatively impacted by the very shallow pool of negative samples in the dataset.

**Table 6:** Overall segmentation performance summary for the resection cavity in dataset D, for different sets of MR sequences as input.

**Inputs**	**Patient-wise**				**Voxel-wise**				**Object-wise**			
	**Recall**	**Precision**	**Specificity**	**bAcc**	**Dice**	**Recall**	**Precision**	**HD95**	**Dice**	**Recall**	**Precision**	**HD95**
flair	92.50 ± 01.65	87.28 ± 01.58	11.95 ± 03.57	52.23 ± 02.56	65.16 ± 26.04	71.04 ± 27.26	66.57 ± 25.77	23.12 ± 31.69	66.55 ± 25.84	71.24 ± 27.70	68.91 ± 26.32	6.38 ± 6.69
t1c	99.24 ± 00.70	89.83 ± 02.09	16.38 ± 05.72	57.81 ± 02.92	76.23 ± 24.79	79.81 ± 24.40	77.06 ± 25.82	13.86 ± 26.75	77.93 ± 23.88	80.01 ± 24.75	80.55 ± 23.69	4.54 ± 5.49
+t1w	99.24 ± 00.30	90.36 ± 02.07	20.94 ± 06.41	60.09 ± 03.07	76.60 ± 24.16	79.94 ± 23.56	77.54 ± 25.52	13.43 ± 27.60	78.40 ± 23.07	80.15 ± 23.88	81.29 ± 22.89	4.44 ± 5.16
+t1d	98.71 ± 00.72	90.84 ± 01.80	25.70 ± 05.02	62.20 ± 02.25	76.42 ± 24.47	79.84 ± 24.12	77.40 ± 25.56	12.76 ± 25.96	77.90 ± 23.69	80.06 ± 24.39	81.26 ± 22.63	4.55 ± 5.42
+flair	98.96 ± 00.31	90.33 ± 01.58	23.83 ± 09.12	61.40 ± 04.55	76.33 ± 24.72	79.46 ± 24.52	78.35 ± 25.25	12.10 ± 21.87	77.96 ± 23.79	79.72 ± 24.65	81.77 ± 22.85	4.78 ± 5.95
+t2w	99.44 ± 00.46	90.29 ± 01.68	24.31 ± 08.09	61.88 ± 04.05	77.28 ± 23.70	80.82 ± 23.37	78.67 ± 24.37	10.45 ± 22.12	78.79 ± 22.91	81.03 ± 23.75	81.86 ± 22.35	4.63 ± 5.96

**Figure 6:**
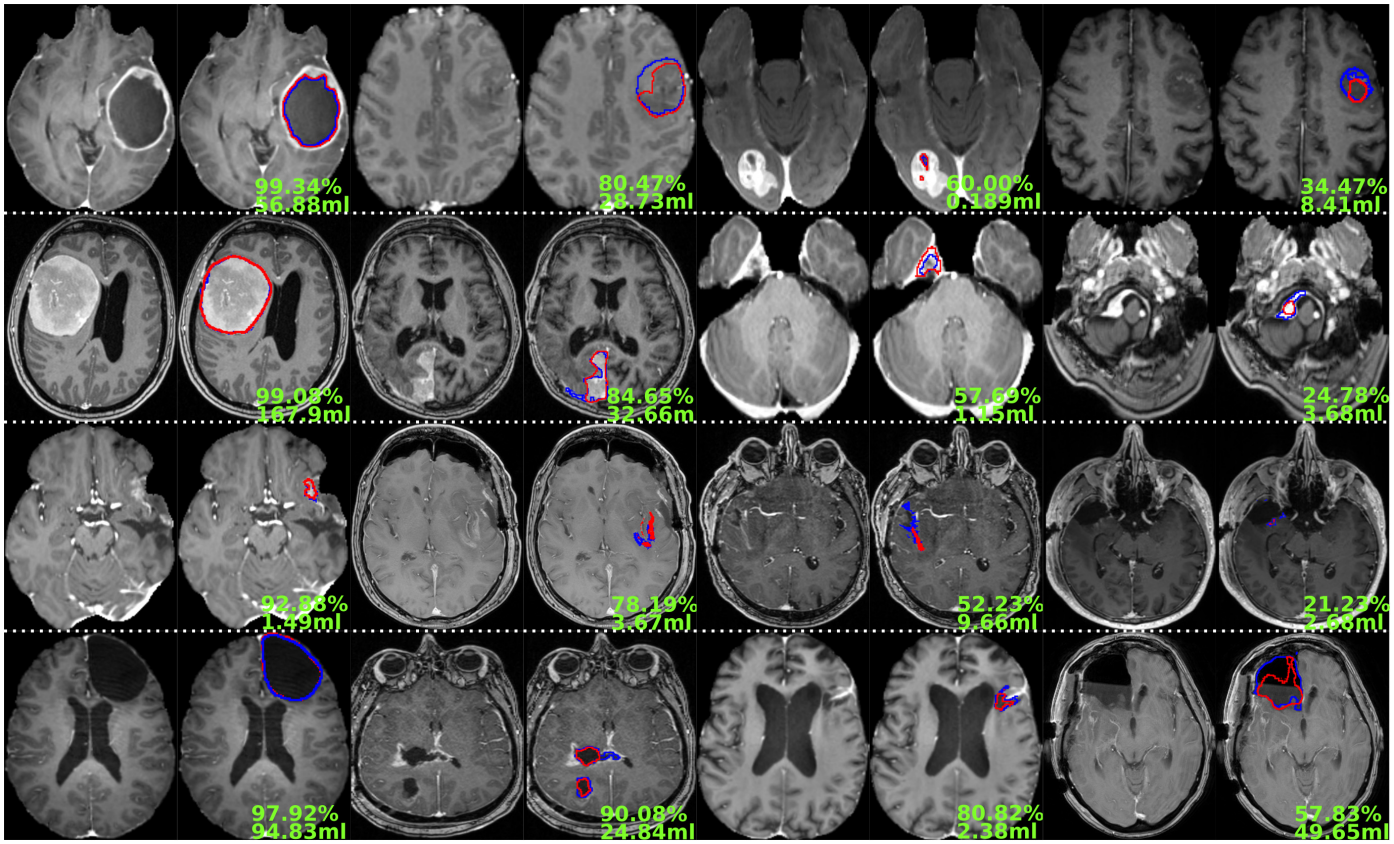
Illustration showing the ground truth (in blue) against the produced prediction (in red) for the NETC, tumor core, residual tumor, and resection cavity from top to bottom. The resulting Dice score and total volume to segment are given in green (image best viewed digitally and in color).

An overview of the models' performance for each structure of interest is provided in Figure 6, showing examples of best to worse results from left to right. Supplementary Figure S5 is a magnified version focusing on the region of interest for each case. For large tumors with a clear contrast-enhancing rim, the NETC model segmented almost perfectly with pixel-wise Dice scores above 95%. When the rim is not visible, potentially in presence of low-/non-contrast-enhancing tumors, the model tended to struggle. Regarding the contrast-enhancing tumor core segmentation model, clearly defined CNS tumors were almost perfectly segmented regardless of size (i.e., glioblastoma or metastasis). Identified cases of struggle often exhibit CNS tumors in unusual location (e.g., around the brain stem) not featured enough in the dataset. Next, the postoperative residual tumor model faced the challenge of segmenting small, fragmented, and not clearly confined structures. Oftentimes only part of the residual tumor was correctly segmented, omitting other smaller components around the cavity. The larger pieces of non-resected tumor, similar to the contrast-enhancing tumor core, were more easily segmented. Finally, the resection cavity segmentation model was more deficient when presented with inhomogeneous cavities displaying varying intensity levels (cf. right-most example in the last row of the figure).

### 5.4 Segmentation performances analysis across cohorts and BraTS challenge benchmarking

The comparison of the contrast-enhancing tumor core segmentation performances, against our previous baseline and the latest BraTS challenge results from 2024, is compiled in Table 7. In our previous study, a specific tumor core segmentation was trained for each tumor type, while a unified model is proposed in this article for all contrast-enhancing tumors. For both the glioma and meningioma groups, the voxel-wise performances have improved from training a unified model, with a Dice score gain of 5% and 2%. The results over the glioma category are quite meaningful to interpret, given the relative high magnitude of both datasets with more than 2, 000 patients included. However, the difference in dataset size for both the meningioma and metastasis categories makes for a difficult direct comparison, as tumors' expression and variability might greatly differ.

**Table 7:** Preoperative contrast-enhancing tumor core segmentation performances, compared to the latest BraTS challenge performances and our previous baseline (25).

**Fold**	**# Samples**	**Patient-wise**				**Voxel-wise**				**Object-wise**			
		**Recall**	**Precision**	**Specificity**	**bAcc**	**Dice**	**Recall**	**Precision**	**HD95**	**Dice**	**Recall**	**Precision**	**HD95**
GLI	3,700	99.40 ± 00.34	99.86 ± 00.12	50.15 ± 44.72	74.78 ± 22.42	89.44 ± 12.40	90.16 ± 12.20	90.77 ± 12.17	4.29 ± 10.19	88.73 ± 12.41	89.30 ± 13.01	90.40 ± 12.52	2.87 ± 3.75
BraTS leaderboard	219	-	-	-	-	-	-	-	-	87.0	-	-	-
Baseline	2,134	-	-	-	-	86.63 ± 12.41	98.32 ± 01.10	95.35 ± 02.13	-	-	85.06 ± 07.87	91.96 ± 03.33	-
MEN	2,278	96.92 ± 00.46	99.95 ± 00.09	94.75 ± 10.00	95.84 ± 04.97	88.83 ± 13.62	88.49 ± 15.46	91.34 ± 11.46	7.93 ± 20.55	89.26 ± 12.90	88.52 ± 15.52	92.26 ± 09.88	2.42 ± 3.91
BraTS leaderboard	70	-	-	-	-	-	-	-	-	84.9	-	-	-
Baseline	719	-	-	-	-	86.62 ± 14.54	94.90 ± 03.29	92.32 ± 04.32	-	-	88.36 ± 03.33	84.32 ± 07.99	-
MET	1,214	99.74 ± 00.20	98.99 ± 01.01	75.99 ± 23.59	87.86 ± 11.73	85.68 ± 14.30	84.28 ± 16.13	89.61 ± 12.51	10.36 ± 21.72	84.94 ± 18.38	86.37 ± 14.27	90.87 ± 11.49	1.48 ± 1.30
BraTS leaderboard	88	-	-	-	-	-	-	-	-	81.0	-	-	-
Baseline	396	-	-	-	-	88.58 ± 14.08	97.73 ± 02.09	95.61 ± 03.46	-	-	82.94 ± 05.13	92.67 ± 05.58	-

Segmentation performances of postoperative contrast-enhancing residual tumors across cohorts, with more than 100 samples, have been reported in Table 8. For fair comparison with the BraTS challenge, performances are reported for the model trained using all four MR sequences as input. A complete report over all cohorts is available in the Supplementary Table S8). A direct comparison with the results from the BraTS 2024 challenge is not possible since the test set is not publicly available. As a result, the latest official leaderboard results over the validation set (available on Synapse), were used in the table for benchmarking purposes. An average lesion-wise Dice score of 76.3% was obtained from the best-performing team for the enhancing tissue category, computed over 188 samples. From our model, averaged over the 1,316 available samples from the BraTS challenge, an object-wise Dice score of 79.7% is obtained, hence on-par with the state-of-the-art. However, large performance variations can be noticed between the main dataset's cohorts. The pixel-wise Dice score decreases to 61.6% for the STO cohort and goes further down to 47% over the SUH cohort. A similar trend is visible across the different cohorts for the patient-wise and object-wise metrics. The performance drop seems to be highly correlated with the average residual tumor volume over each cohort (cf. Supplementary Table S3) going from 15 ml over the BraTS cohort down to 3.7 ml for the SUH cohort.

**Table 8:** Contrast-enhancing tumor segmentation performances for cohorts with at least 100 patients, compared to the latest BraTS challenge performances, using all four input sequences.

**Fold**	**# Samples**	**Patient-wise**				**Voxel-wise**				**Object-wise**			
		**Recall**	**Precision**	**Specificity**	**bAcc**	**Dice**	**Recall**	**Precision**	**HD95**	**Dice**	**Recall**	**Precision**	**HD95**
STO	407	94.69 ± 01.36	71.49 ± 03.96	59.75 ± 09.61	77.22 ± 04.28	61.64 ± 23.10	64.41 ± 27.70	68.10 ± 24.26	10.77 ± 14.14	61.86 ± 23.16	67.37 ± 27.23	73.52 ± 20.85	6.44 ± 6.24
SUH	199	77.18 ± 06.07	87.91 ± 08.23	84.51 ± 09.20	80.84 ± 04.68	47.16 ± 30.60	50.90 ± 34.51	51.02 ± 33.20	9.01 ± 12.05	49.67 ± 30.73	54.19 ± 34.84	74.37 ± 23.22	4.14 ± 5.11
BraTS	1316	98.36 ± 01.36	89.42 ± 03.67	77.88 ± 05.55	88.12 ± 02.91	80.27 ± 19.36	81.71 ± 19.13	82.44 ± 21.08	5.93 ± 10.91	79.77 ± 20.51	82.19 ± 20.48	83.42 ± 20.04	3.25 ± 4.42
BraTS leaderboard	188	-	-	-	-	-	-	-	-	76.30	-	-	

For the resection cavity, performances over cohorts with at least 100 samples are reported in Table 9, using the same method as described above to benchmark against the BraTS challenge 2024. A complete report over all cohorts is available in the Supplementary Tables S9, S10). An average lesion-wise Dice score of 71.5% was obtained from the best-performing team for the resection cavity segmentation. From our best model over the BraTS cohort, an object-wise Dice score of 76.33% is obtained. The inter-cohort variability is much lower with average Dice scores around 80%. Interestingly, as reported in Supplementary Table S4, average resection cavity volumes are relatively similar across all cohorts, at around 20 ml.

**Table 9:** Resection cavity segmentation performances for cohorts with at least 100 patients, compared to the latest BraTS challenge performances, using all four input sequences.

**Cohort**	**# Samples**	**Patient-wise**				**Voxel-wise**				**Object-wise**			
		**Recall**	**Precision**	**Specificity**	**bAcc**	**Dice**	**Recall**	**Precision**	**HD95**	**Dice**	**Recall**	**Precision**	**HD95**
STO	275	99.64 ± 00.85	100.00 ± 00.00	100.00 ± 00.00	99.82 ± 00.43	81.63 ± 17.51	84.71 ± 17.94	81.65 ± 18.77	14.55 ± 33.81	83.59 ± 16.66	84.53 ± 18.16	85.59 ± 15.74	4.39 ± 5.08
SUH	165	100.00 ± 00.00	98.18 ± 02.69	58.18 ± 48.99	79.09 ± 24.49	76.02 ± 22.89	81.34 ± 22.14	76.14 ± 23.77	10.59 ± 25.77	77.97 ± 21.60	81.62 ± 22.28	79.25 ± 21.56	3.49 ± 5.96
BraTS	1316	99.55 ± 00.29	86.53 ± 02.06	19.58 ± 06.53	59.57 ± 03.16	75.18 ± 25.78	79.03 ± 25.22	77.00 ± 26.50	9.09 ± 14.73	76.33 ± 25.49	79.78 ± 25.40	79.98 ± 25.01	4.92 ± 6.16
BraTS leaderboard	188	-	-	-	-	-	-	-	71.50	-	-		

### 5.5 (v) Generalizability investigation

For the STO cohort, available metadata included MR scanner manufacturer, scanner type, acquisition protocol, and field strength (cf. Supplementary Tables S5, S6). Across these metadata categories, no significant differences in segmentation performance were observed between the main groups (see Supplementary Tables S13–S20). This suggests proper generalizability of the trained models despite variability in MRI acquisition protocols.

**Figure 7:**
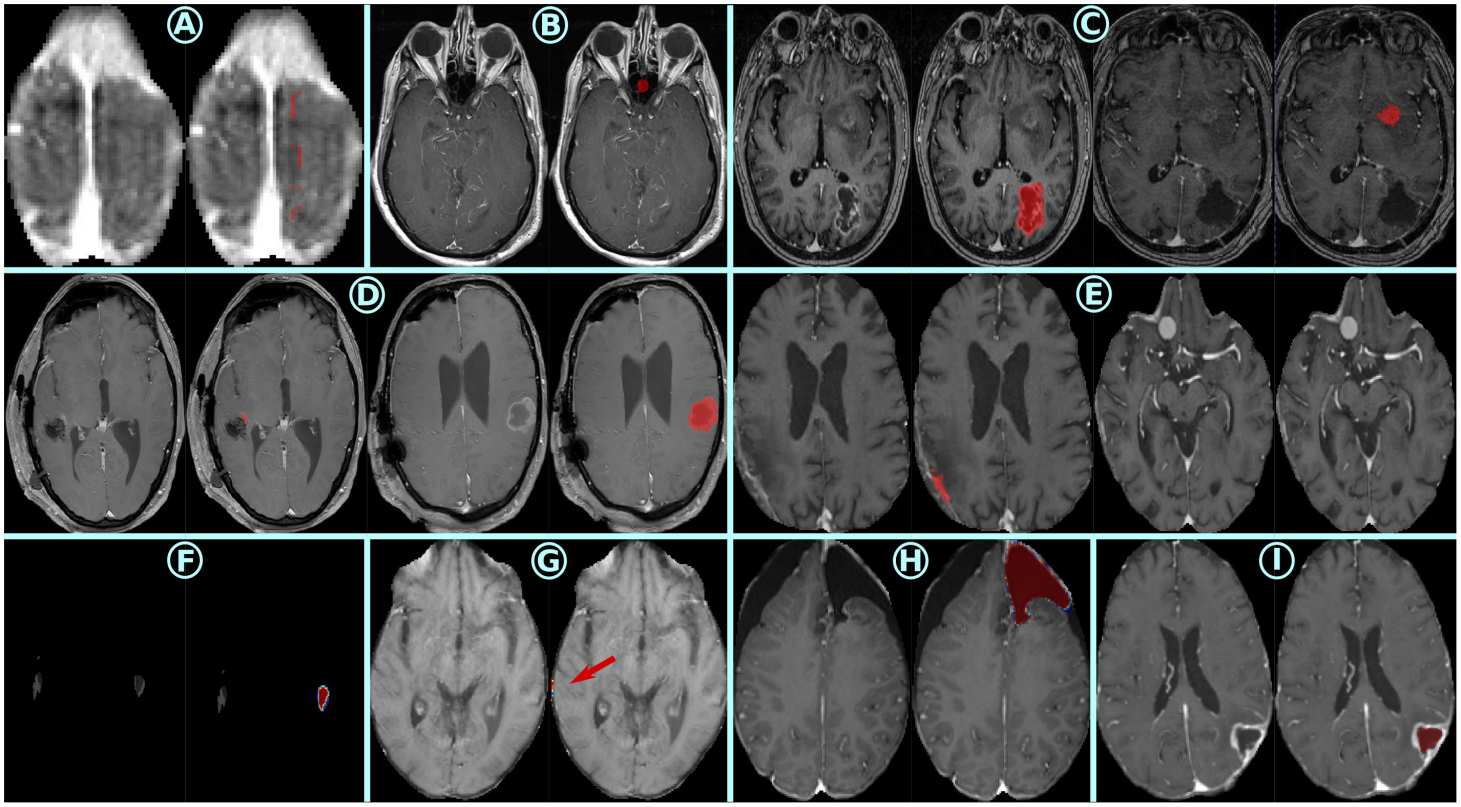
Cases where the models exhibited poor performance metrics, each separated by light blue lines and assigned a blue letter **(A-I)**. In the first two rows, the ground truth is represented in red. In the bottom row, the model predictions are represented with a color map.

A visual inspection of cases where the different models provided poor metrics was performed and typical examples have been assembled in Figure 7. Cases A and B illustrate ground truth noise, likely due to semi-automatic annotations that were not fully corrected. Case C shows a postoperative annotation performed on a different tumor than the surgical target, leading to erroneous residual tumor assessment. Case D depicts a residual tumor correctly annotated, yet another tumor region was also fully segmented (i.e., mix of ET and NETC classes). Case E represents a scenario where the residual tumor has been correctly annotated but other contrast-enhancing regions untargeted by surgery were omitted. Together, cases C, D, and E highlight inconsistencies common in multi-purpose datasets, where annotators and protocols vary across institutions and tasks. Regarding model limitations, cases F and G demonstrate pitfalls of skull-stripped inputs, where atypical brain borders caused confusion due to abnormal gradients and contrast. Finally, case H features RC model predictions over a likely old resection cavity not marked in the ground truth, while case I highlights RC model over-segmentation of NETC regions in tumor areas not targeted by surgery. Additional examples can be accessed in the Supplementary Figures S6–S8).

For patient-wise classification metrics, a Dice threshold of 0.1% is extremely permissive to categorize true positive cases. As reported in Table 10, both patient-wise recall and precision values decline progressively with stricter thresholds. At a threshold of 25%, a patient-wise recall of 91.51 and precision of 82.72 are still relatively close to the values obtained with the most permissive threshold. Higher thresholds substantially reduce false positives but at the cost of missing many true positives. Yet, using Dice score as detection threshold should be interpreted with care and in relation to object size. For small structures such as residual tumor averaging 5 ml (i.e., 5, 000 voxels), a 50% Dice score does not necessarily indicate poor performance. For example, this could correspond to only 1, 000 voxels being misclassified, often reflecting minor boundary discrepancies rather than clinically meaningful errors.

**Table 10:** Sensitivity analysis results showing the impact of varying detection thresholds on overall performances for the ET segmentation model using all input sequences.

**Threshold (%)**	**# Samples**			**Patient-wise**			
	**Total**	**Pos**.	**TP**	**Recall**	**Precision**	**Specificity**	**bAcc**
0.1	2,224	1,375	1,314	95.54 ± 00.70	84.06 ± 02.56	70.61 ± 04.78	83.08 ± 02.48
10	2,224	1,375	1,296	94.23 ± 00.82	83.89 ± 02.51	70.61 ± 04.78	82.42 ± 02.17
25	2,224	1,375	1,259	91.51 ± 01.14	82.72 ± 02.63	68.92 ± 05.22	80.21 ± 02.35
50	2,224	1,375	1,119	81.39 ± 01.36	81.83 ± 02.62	70.61 ± 04.78	76.00 ± 01.99
75	2,224	1,375	793	57.56 ± 01.34	77.38 ± 03.87	72.63 ± 05.08	65.10 ± 02.75

## 6 Discussion

This study presents a comprehensive investigation into standardized pre- and post-surgical automatic assessment reporting for central nervous system tumors. First, unified segmentation models for both preoperative contrast-enhancing and non-enhancing tumor core structures were introduced, using the Attention U-Net architecture. Second, postoperative segmentation models for contrast-enhancing residual tumor and resection cavity were developed, leveraging the same architecture with variations in MR sequence inputs. Finally, classification models for MR sequence identification and tumor type differentiation were explored using DenseNet. The primary contribution of this study is achieving state-of-the-art segmentation performance, validated against the latest BraTS challenge results. Building on these models, an automated surgical reporting pipeline was proposed, aligned with the latest RANO 2.0 guidelines. Lastly, all models and methods are integrated into the open-access Raidionics software, providing a standardized reporting solution for clinical use.

The preoperative contrast-enhancing tumor core segmentation dataset includes a large and diverse patient population, covering all major CNS tumor subtypes. For contrast-enhancing tumors, the t1c MR sequence remains the most informative and critical modality. A key limitation, however, is the inconsistent availability of additional MR sequences across patients, except in the BraTS cohort. In clinical practice, multiple sequences (i.e., t1c, t1w, and flair) are essential for optimal treatment planning and prognosis. However, since all four standard sequences are not always available at every center, developing models able to perform well using a single sequence has high practical value. To create unified segmentation models applicable to all CNS tumor types, the tumor core and non contrast-enhancing tumor core structures were considered separately in datasets A and B. This choice reflects the significant under-representation of necrotic and cystic regions in meningiomas and metastases, which would otherwise introduce class imbalance and complexity in a mixed model. For postoperative segmentation of contrast-enhancing residual tumor, incorporating multiple sequences becomes imperative. For example, separating blood products from residual tumor requires looking at both t1c and t1w scans. To enhance contrast differences and reduce confusion, using t1 subtraction (i.e., t1d) as additional input was experimented but did not provide meaningful impact. Alternatively, Diffusion-Weighted Imaging (DWI) and Apparent Diffusion Coefficient (ADC) maps could be considered as input sequences postoperatively in the future. Both sequences could help further disambiguate residual disease, exhibiting restricted diffusion, from hemorrhage and infarction. However, these sequences are not consistently acquired, and their integration would required robust handling of missing modalities. In dataset C where sequence availability varies, increasing required input modalities reduces usable cases. Yet, sufficient data remains to train effective models across all configurations. Dataset D is an exception, containing a higher proportion of non contrast-enhancing cases. Mixing contrast-enhancing and non contrast-enhancing samples supports the goal of unified models. Finally, a resection cavity model operating primarily on FLAIR scans is essential for complete surgical reporting across all major CNS tumor types, especially if t1 sequences are unavailable. To expand training for this configuration, BraTS cases were included, originally segmented on t1c then co-registered to FLAIR. Notably, resection cavities are often undefined for meningiomas, as these tumors lie outside of the brain.

MR sequence classification achieved near-perfect performance, with only 32 misclassifications out of 8, 000 cases. Each error was linked to acquisition issues such as motion or illumination artifacts. Even when correctly classified, scans with severe artifacts may still be unsuitable for reliable segmentation. On the other hand, tumor type classification exhibited limitations, reflected by the lower balanced accuracy of 85%. The decision to use only t1c as input was driven by dataset constraints, restricting the model to morphological cues which are insufficient for robust differentiation. Integrating anatomy-guided inputs or leveraging the high-performing tumor core segmentation model should direct the network's attention to relevant regions and subtle intra-tumoral patterns (e.g., rim thickness, necrosis). Additionally, incorporating complementary sequences, such as FLAIR and T2 when available, could enhance classification performance and enable unified models for both contrast-enhancing and non-enhancing tumors.

For preoperative tumor core segmentation of contrast-enhancing CNS tumors, the unified model outperformed type-specific models, surpassing both our previous results and latest BraTS challenge leaderboards. Using only t1c input does not appear to be a limitation, as performance was on par or higher than BraTS results obtained with all four sequences. Postoperatively, the contrast-enhancing residual tumor segmentation models performed well, achieving an object-wise Dice score of 70% and a HD95 of 4.32 mm. Including t1w alongside t1c improved object-wise Dice by 3%, and adding flair and t2 yielded another 3% gain. However, these gains must be interpreted with caution due to potential biases introduced by varying sample sizes across configurations. Specifically, a direct comparison of performance across input combinations is limited, as each was evaluated on a different subset of patients. Notably, when all four MR sequences were included, the BraTS cohort constituted 60% of dataset C, introducing potential bias toward its distribution. Fortunately, including t1w reduced the sample size by only 30 patients, making this configuration both clinically meaningful and more representative as t1w helps distinguish residual tumor from postoperative blood products. In a previous work (16), an ablation study to assess the isolated impact of each sequence on overall performances was performed, over dataset C excluding the BraTS cohort. The results indicated a similar trend with improved performance from using t1w in addition to t1c as input, while flair and t2w inclusion had limited impact. For resection cavity segmentation, incremental sequence inclusion had minimal effect, with stable object-wise Dice scores around 78%. The specific inclusion of t1d inputs did not have a noticeable impact on overall performances. The use of FLAIR sequences only yielded lower performance, down to 66%. An explanation might come from the type of CNS tumor featured in the different cohorts. Dataset D differs from others by including more non contrast-enhancing tumors, which may explain some variability. The models' classification ability, reflected in the patient-wise metrics, should be interpreted with care. Most cohorts have highly skewed positive-to-negative ratio whereby nearly all patients exhibit the structure of interested to be segmented. As such, low specificity and balanced accuracy values are almost inevitable, strongly influenced by the data distribution. In this context, patient-wise recall and precision provide a more meaningful assessment, particularly from a clinical perspective. Ensuring all patients with tumor are detected (i.e., recall) while minimizing false positives (i.e., precision) is critical. Missing a tumor region carries greater risk than over-segmentation. Specific model training techniques, known to handle better large variations in sample distribution or class imbalance (e.g., bootstrapping, hard-mining), could be investigated. Alternatively, architectures able to perform classification and segmentation at the same time (e.g., Mask R-CNN) could help mitigate this issue.

Postoperative imaging is inherently complex, many structures are fragmented, making reliance on a single metric potentially misleading. Multi-metric validation combining patient-wise, voxel-wise, and object-wise analyses provides a more comprehensive understanding of model performance, capturing both fine-grained segmentation accuracy and clinically relevant detection capabilities. In addition, ground truth inconsistencies are expected in large, multi-institutional datasets making it challenging to achieve pixel-perfect annotations. While such noise is generally tolerable for training robust models, some voxel-wise metrics might be inflated (e.g., HD95 as highlighted in Supplementary Figure S6). Object-wise metrics which discard clinically irrelevant fragments below a predefined threshold, as done in the BraTS challenge, provide a more stable and clinically meaningful assessment. Adaptive ROI-based voxel-wise metrics computation could be investigated, focusing on regions near the preoperative tumor site targeted for surgery.

Analysis of inter-cohort variability reveals several factors influencing residual tumor segmentation performance. Notably, as observed in the per-cohort average volumes, a substantial difference between BraTS and other cohorts exists with structures being nearly twice as large. Such volume discrepancy likely explains the 20% Dice score gap, as larger targets are generally easier to segment. Another key distinction is postoperative imaging timing during patient care. While all other cohorts include only early postoperative scans, BraTS comprises multiple postoperative time points. Scans acquired several months after surgery typically exhibit reduced cavity content and resolution of surgical blood products, making residual tumor boundaries more discernible. These differences underscore the significant variability in MR imaging across institutions and highlight the importance of diverse, multi-institutional datasets for training models with strong generalizability. Balancing data quality and quantity remains particularly challenging for residual tumor segmentation. Additional variability may stem from differences in surgical practices (e.g., intraoperative decisions, extent of resection) and treatment strategies (e.g., surgery, chemotherapy). This interpretation is reinforced by the more consistent performance observed across cohorts for resection cavity segmentation, suggesting that this task is less affected by clinical variability.

A direct and exact comparison with BraTS challenge performance is not feasible due to the unavailability of their test sets and potential differences in design choices. Specifically, the implementation details of metric computation can significantly impact the results, most notably in the pairing strategy to compute object-wise metrics (referred to as lesion-wise in BraTS). Nonetheless, we believe that our own implementation provides a fair and consistent assessment of model performance. A key source of variation lies in the threshold used to determine whether a model prediction is considered correct, either on a patient-wise or object-wise basis. For instance, adopting a very low Dice threshold (e.g., 0.1%) between ground truth and detection favors patient-wise recall but results in lower average Dice scores. Conversely, using a stricter threshold such as 50% Dice aligns more closely with computer vision conventions, yielding improved pixel-wise and object-wise performance but at the cost of reduced sensitivity. While this stricter threshold may reduce the patient-wise recall, it arguably better reflects clinical relevance through qualitative visual agreement. For more robust statistical comparisons across models, limiting the analysis to patients with all four MR sequences would be a more rigorous choice. However in dataset A, excluding several hundred cases would reduce tumor heterogeneity and introduce a bias toward the predominantly American BraTS cohort.

The proposed standardized surgical report offers an almost complete quantitative depiction of key structures for both contrast-enhancing and non contrast-enhancing tumors. Volumetric assessments of the brain, tumor core, non-enhancing tumor core, resection cavity, and FLAIR hyperintensity enable evaluations aligned with RANO 2.0 guidelines for CNS tumors. The inclusion of MR sequence and tumor type classification models in the reporting pipeline also alleviates the burden for the user. The reporting process requires only a folder containing MR acquisitions organized into preoperative and postoperative scans. Segmentation of the most relevant structures in contrast-enhancing and non contrast-enhancing CNS tumors is fully supported. A fine-grained tumor subtype classification or image-based biomarker prediction would be interesting to investigate. Furthermore, incorporating segmentation models for post-operative complications, such as hemorrhages or infarctions, remains an important direction for enhancing clinical utility.

Future work could explore the development of models capable of segmenting all relevant structures simultaneously or incorporating global context refinement mechanisms. Although *post-hoc* refinement mitigates inconsistencies, it cannot fully resolve volumetric conflicts arising from independent predictions. Implementing a single unified multi-class model could intrinsically enforce anatomical relationships during training. However, it would likely require a highly curated and specific dataset, which remains challenging outside of the BraTS challenge. In addition, severe class imbalance is problematic, for example with the NETC structure not consistently present across all tumor types. Current state-of-the-art strategies in BraTS adopt an additive approach by segmenting the enhancing tumor, tumor core, and whole tumor classes hence bypassing the challenges from the NETC structure. Additionally, another priority is resolving the ambiguity between resection cavities and NETC regions, which often exhibit similar appearances on t1c. Such issue becomes particularly problematic in re-operation scenarios, where preexisting cavities can lead to inaccurate volumetric measurements in standardized preoperative reports. Since preoperative models are not trained to distinguish such occurrences, employing a STAPLE-based fusion approach, incorporating both preoperative and postoperative segmentation masks, may improve overall accuracy. Another promising direction involves the development of multi-input segmentation models able to accommodate variable combinations of MR sequences (38). Data augmentation techniques, such as randomly masking one or more sequences during training, may improve robustness. To further capitalize on the existing datasets, including patients with missing sequences would be beneficial. In this context, the use of generative diffusion models to synthetize absent MR scans also warrants exploration. Lastly, performing clinical validation of the standardized report is essential. In particular, assessing the predictive value of the automatically derived measurements in relation to clinical outcomes, such as survival and quality of life, will be essential for ensuring the clinical utility and adoption of such tools. A similar approach to Majewska et al. (39) for assessing the prognostic value of automatic extent-of-resection computation will be followed.

## Data Availability

The datasets analyzed in this study can not be made publicly available according to the ethical approval for this study. Requests to access the datasets should be directed to david.bouget@sintef.no. Accession codes for the Raidionics environment with all related information is available at https://github.com/raidionics. More specifically, all trained models can be accessed at https://github.com/raidionics/Raidionics-models/releases/tag/v1.3.0-rc, the Raidionics software can be found at https://github.com/raidionics/Raidionics. Finally, the source code used to compute the validation metrics is available at https://github.com/dbouget/validation_metrics_computation.
