# Supplementary material for: Automatic and standardized reporting of perioperative MRIs in patients with central nervous system tumors
Source: Front Neurol. 2026 Feb 6;16:1707481. doi: 10.3389/fneur.2025.1707481 (PMC12920189; doi:10.3389/fneur.2025.1707481)
Supplement: Supplementary file 1 [file Data_Sheet_1.pdf]

Supplementary Material

1 DATA

1.1 Cohort abbreviations

Abbreviations used for each cohort featured in one of the four datasets used in this study are listed in the following:

1. BraTS - MICCAI BraTS challenges (editions 2023 and 2024)
2. STO - St. Olavs hospital, Trondheim University Hospital, Trondheim, Norway
3. STOP - Polyclinics affiliated to St. Olavs hospital, Trondheim University Hospital, Trondheim, Norway
4. SUH - Sahlgrenska University Hospital, Göteborg, Sweden
5. UCSF - University of California San Francisco Medical Center, U.S.A
6. ETZ - St Elisabeth Hospital, Tilburg, Netherlands
7. VUmc - Amsterdam University Medical Centers, location VU medical center, Netherlands
8. HMC - Medical Center Haaglanden, the Hague, Netherlands
9. HUM - Humanitas Research Hospital, Milano, Italy
10. UMCU - University Medical Center Utrecht, Netherlands
11. ISALA - Isala hospital, Zwolle, Netherlands
12. MUW - Medical University Vienna, Austria
13. UMCG - University Medical Center Groningen, Netherlands
14. PARIS - Hôpital Lariboisière, Paris, France
15. SLZ - Medical Center Slotervaart, Amsterdam, Netherlands
16. NWZ - Northwest Clinics, Alkmaar, Netherlands
17. BOS - Brigham and Women's Hospital, Boston, USA
18. OSL - Oslo University Hospital, Oslo, Norway
19. BOS - Brigham and Women's Hospital, Boston, USA

1.2 Dataset statistics

As part of the following datasets, patients suffering from glioma (GLI), glioblastoma (GBM), meningioma (MEN), and metastasis (MET) have been included. For each dataset, overview tables are reporting average values per cohort and/or CNS tumor type. In addition to total number of samples and average volumes, the total number of positive samples and the positive rate are also provided. Shortened from positive-to-negative sample ratio, the measure indicates the proportion of positive samples, i.e., containing the structure of interest.

Dataset A - Contrast-enhancing tumor core

For dataset A, a detailed cohort-wise and tumor type wise description is presented in Table. S1. The BraTS challenge is by far the biggest contributor across all tumor types, with the St. Olavs hospital placing second. Average volumes are consistent across all cohorts for corresponding tumor types. The biggest CNS tumors being glioblastomas with a volume larger than $30\,ml$ on average. It can be noticed that meningioma patients referred to surgery had much larger tumors on average than patients followed at the outpatient clinic, with a $18\,ml$ difference. A few contrast-enhancing tumors were not formally identified for the STO cohort and were labelled as *Others*. In dataset A, only 61 samples out of 7212 do not contain any contrast-enhancing tumor to segment.

Table S1. Detailed overview of the preoperative tumor core segmentation dataset (dataset A), per cohort and tumor type.

Cohort	Type	Samples	Positives	Positive rate (%)	Volume (ml)
BraTS	GLI	1663	1656	99.57	37.87 ± 32.67
BraTS	MEN	1500	1494	99.60	21.12 ± 29.98
BraTS	MET	817	777	95.10	09.17 ± 12.73
STO	GBM	596	596	100	32.93 ± 32.02
STOP	MEN	442	440	99.54	12.34 ± 21.72
STO	MEN	336	336	100	30.81 ± 34.70
STO	MET	332	332	100	19.79 ± 18.57
SUH	GBM	251	251	100	35.46 ± 28.19
UMCU	GBM	171	171	100	36.82 ± 27.96
ETZ	GBM	153	153	100	36.83 ± 28.86
UCSF	GBM	134	133	99.25	28.69 ± 26.83
HMC	GBM	103	103	100	42.17 ± 29.45
VUmc	GBM	97	97	100	31.52 ± 24.01
UMCG	GBM	86	86	100	32.26 ± 27.86
MUW	GBM	83	83	100	34.35 ± 29.33
HUM	GBM	75	75	100	26.71 ± 22.26
PARIS	GBM	74	72	97.29	32.44 ± 23.67
ISALA	GBM	72	72	100	37.62 ± 31.18
OUS	MET	67	64	95.52	06.27 ± 07.18
SLZ	GBM	49	49	100	36.87 ± 28.38
NWZ	GBM	38	38	100	26.47 ± 23.76
STO	Others	28	28	100	21.20 ± 20.04

Dataset B - Non-enhancing tumor core

For dataset B, a detailed cohort-wise and tumor type wise description is presented in Table. S2. For the glioma category, a distinction is made between preoperative and postoperative acquisitions, while only preoperative data are available for the other two tumor types. The BraTS challenge is the only contributor, and the NETC structure is predominantly featured in preoperative gliomas, with a 95% occurrence rate and

Table S2. Detailed overview of the NETC segmentation dataset (dataset B). Preoperative data are indicated with preop. and postoperative data with postop.

Cohort	Type	Samples	Positives	Positive rate (%)	Volume (ml)
BraTS	GLI preop.	1294	1224	94.59	15.61 ± 21.94
BraTS	GLI postop.	1316	492	37.38	04.56 ± 06.92
BraTS	MEN	1000	343	34.30	03.18 ± 08.87
BraTS	MET	817	448	54.83	03.64 ± 06.77

15 ml average volume. For the other groups, NETC is only featured with a 40% positive rate and a much lower average volume around 3.5 ml.

Dataset C - Residual tumor (enhancing tissue)

For dataset C, a detailed cohort-wise description is presented in Table. S3. The rate of positive to negative samples is highly varying from cohort to cohort, from 34% at the lowest up to 74%. On average, residual tumor volumes are similar across all but one cohort, ranging from 3 ml to 6 ml. The BraTS challenge represents an exception where the samples exhibit residual tumor with an average volume of 15 ml.

Table S3. Detailed overview of the contrast-enhancing residual tumor dataset per cohort (dataset C).

Cohort	Samples	Positives	Positive rate (%)	Volume (ml)
BraTS	1316	875	66.48	15.25 ± 21.41
STO	421	220	52.25	6.35 ± 8.18
SUH	200	119	59.50	3.67 ± 5.72
UCSF	109	81	74.31	4.92 ± 9.11
ETZ	101	73	72.27	4.94 ± 5.19
VUmc	74	52	70.27	2.74 ± 3.86
HMC	67	43	64.18	5.06 ± 6.38
HUM	56	35	62.50	3.70 ± 3.90
UMCU	54	21	38.88	4.27 ± 4.91
ISALA	52	18	34.61	3.86 ± 6.75
MUW	51	35	68.62	3.91 ± 5.94
UMCG	48	29	60.41	4.52 ± 5.30
PARIS	43	31	72.09	4.37 ± 4.82
SLZ	28	21	75.00	3.84 ± 5.41
NWZ	27	21	77.77	3.76 ± 4.69

Dataset D - Resection cavity

For dataset D, a detailed cohort-wise description is presented in Table. S4. For all cohorts but BraTS, a resection cavity is present for each sample with an almost 100% positive rate. For the BraTS cohort, around 18% of all samples do not feature a resection cavity. For the three most-populated cohorts, an average resection cavity volume of 16 ml is measured. Some of the less populated cohorts do exhibit average volumes up to 30 ml. The BOS cohort is comprised solely of non-contrast-enhancing tumors, a mixture of contrast-enhancing (322) and non-contrast-enhancing (74) tumors is present in the STO cohort, and the BraTS cohort also includes a few non-contrast-enhancing tumors.

Table S4. Detailed overview of the resection cavity segmentation dataset per cohort (dataset D).

Cohort	Samples	Positives	Positive rate (%)	Volume (ml)
BraTS	1316	1092	82.97	16.71 ± 22.09
STO	396	396	100	17.38 ± 13.59
BOS	236	235	99.57	15.07 ± 16.29
SUH	167	164	98.20	22.09 ± 18.61
ETZ	57	57	100	21.72 ± 15.62
ISALA	16	16	100	31.21 ± 20.75
VUMC	16	16	100	20.97 ± 12.05
MUW	15	15	100	20.97 ± 14.68
UMCG	12	12	100	17.97 ± 11.92
UCSF	10	10	100	29.70 ± 16.96
HMC	9	9	100	20.97 ± 18.89
PARIS	9	9	100	30.20 ± 11.49
UMCU	9	9	100	26.04 ± 13.28
HUM	4	4	100	30.87 ± 24.63
SLZ	4	4	100	28.39 ± 25.12
NWZ	3	3	100	14.49 ± 04.18

1.3 Detailed metadata analysis for the STO cohort

An analysis of the available metadata for the STO cohort and GBM tumor type was conducted, focusing on the $t1c$ MR scans. The metadata contained information about: scanner manufacturer, scanner model, field strength, image frequency, and scanning sequence used. The preoperative acquisition conditions are detailed in Table S5, while the postoperative acquisition protocols are detailed in Table S6. The following scanning sequence abbreviations are used: gradient-echo scanning sequence with inversion recovery (noted GR/IR), gradient-echo scanning sequence (noted GR), spin echo (noted SE), and inversion recovery spin echo (noted SE/IR). For the 595 patients with preoperative metadata, Siemens scanners were used for more than 80% of all acquisitions. The field strength distribution was close to even between 1.5T and 3T use, with only 1% of acquisition performed with a 1T scanner. The GR/IR scanning sequence was also predominantly used (79.1%), favored with Siemens scanners.

Table S5. Detailed summary of acquisition conditions for the STO cohort from dataset A, including only glioblastoma patients.

Manufacturer	Scanner model	Field strength	Image frequency	Scanning sequence
Siemens (490)	Avanto (212)	1.5T	64	GR/IR (205)<br>GR (2)<br>SE (4)<br>SE/IR (1)
	Avanto fit (20)	1.5T	64	GR/IR
	TrioTim (17)	3T	123	GR/IR (16)<br>GR (1)
	Skyra (126)	3T	123	GR/IR
	Prisma (77)	3T	123	GR/IR (74)<br>SE (2)<br>GR (1)
	Biograph mMR (17)	3T	123	GR/IR
	Aera (4)	1.5T	64	GR/IR (3)<br>SE (1)
	Symphony (10)	1.5T	64	GR/IR (8)<br>SE (2)
	Magnetom Sola (5)	1.5T	64	GR
	Sonata (1)	1.5T	64	SE
	OsiriX (1)	3T	123	GR/IR
Philips Medical System (82)	Intera (64)	3T (55)	128	GR
		1T (6)	43	SE (5)<br>GR (1)
		1.5T (3)	64	GR (2)<br>SE (1)
	Achieva (12)	1.5T	64	GR (10)<br>SE (2)
	Achieva dStream (1)	1.5T	64	GR
	Ingenia (4)	1.5T	64	SE (3)
			64	GR (1)
	Ingenia Evolution (1)	1.5T	64	SE
GE Medical Systems (23)	Signa HDXt (14)	1.5T	64	GR (10)<br>SE (2)<br>SE/IR (2)
	Signa HDX (5)	1.5T	64	SE (4)<br>SE/IR (1)
	Architect (2)	3T	128	GR/IR (1)<br>GR (1)
	Genesis Signa (2)	1.5T	64	SE

For the 412 patients with postoperative metadata, the same conclusions can be drawn with Siemens scanners predominantly used, an equal distribution between 1.5T and 3T field strength scanners, and GR/IR as main scanning sequence.

Table S6. Detailed summary of acquisition conditions for the STO cohort from dataset C.

Manufacturer	Scanner model	Field strength	Image frequency	Scanning sequence
Siemens (379)	Avanto (182)	1.5T	64	GR/IR (178)<br>GR (2)<br>SE (2)
	Avanto fit (40)	1.5T	64	GR/IR
	TrioTim (15)	3T	123	GR/IR (12)<br>GR (2)<br>SE (1)
	Skyra (52)	3T	123	GR/IR (50)<br>GR (2)
	Prisma (82)	3T	123	GR/IR (76)<br>GR (6)
	Magnetom Sola (8)	1.5T	64	GR
Philips Medical System (33)	Intera (32)	3T	128	GR
	Achieva (1)	1.5T	64	GR

2 RESULTS

2.1 Preoperative tumor core and NETC segmentation performances

A detailed cohort-wise performance summary is presented in Table. S7 for the preoperative contrast-enhancing tumor core segmentation, over t1c MR scans from dataset A. The classification ability of the model is perfect for half the cohorts and not dropping under 97% for the other half, in terms of recall and precision. Since the positive rate in dataset A lies around 99%, the specificity and balanced accuracy values are drastically lowered when a few negative patients are misclassified. Regarding the segmentation performance, the object-wise Dice scores span from 86% up to 91% for all samples where the patient was given surgical treatment, indicating a strong ability to generalize. For the STOP cohort, the average object-wise Dice score was slightly lower at 84%, for patients followed at the outpatient clinic, with one of the lowest average tumor volume.

Table S7. Preoperative contrast-enhancing tumor core segmentation performances, over t1c MR scans from dataset A, per cohort.

		Patient-wise				Voxel-wise			Object-wise		
Cohort	# Samples	Recall	Precision	Specificity	bAcc	Dice	Recall	Precision	Dice	Recall	Precision
ETZ	153	100.00 ± 00.00	100.00 ± 00.00	100.00 ± 00.00	100.00 ± 00.00	89.98 ± 09.76	92.20 ± 10.52	88.63 ± 10.62	88.18 ± 10.90	91.17 ± 11.51	86.96 ± 11.78
HMC	103	100.00 ± 00.00	100.00 ± 00.00	100.00 ± 00.00	100.00 ± 00.00	90.25 ± 08.58	92.43 ± 09.20	88.56 ± 09.61	89.62 ± 08.78	91.92 ± 10.04	88.53 ± 07.24
HUM	75	100.00 ± 00.00	100.00 ± 00.00	100.00 ± 00.00	100.00 ± 00.00	90.33 ± 06.41	93.93 ± 04.89	87.59 ± 09.25	89.35 ± 06.68	93.44 ± 05.67	86.61 ± 09.79
ISALA	72	100.00 ± 00.00	100.00 ± 00.00	100.00 ± 00.00	100.00 ± 00.00	90.60 ± 08.25	88.39 ± 10.59	93.60 ± 06.57	90.77 ± 07.22	88.65 ± 08.65	93.65 ± 07.39
BraTS	3980	98.94 ± 00.13	99.59 ± 00.09	40.82 ± 16.85	69.88 ± 08.41	86.93 ± 17.91	85.99 ± 18.79	90.26 ± 16.63	86.43 ± 19.46	86.57 ± 18.46	91.59 ± 14.21
MUW	83	100.00 ± 00.00	100.00 ± 00.00	100.00 ± 00.00	100.00 ± 00.00	89.13 ± 10.52	94.04 ± 06.74	86.25 ± 11.97	88.62 ± 06.99	92.82 ± 08.66	86.22 ± 08.92
NWZ	38	100.00 ± 00.00	100.00 ± 00.00	100.00 ± 00.00	100.00 ± 00.00	87.40 ± 11.87	91.75 ± 11.47	84.71 ± 13.24	88.26 ± 08.14	93.50 ± 06.62	84.99 ± 11.59
OUS	67	98.51 ± 05.00	98.51 ± 04.00	85.07 ± 40.00	91.79 ± 19.53	90.18 ± 05.68	88.41 ± 06.59	92.56 ± 07.09	87.31 ± 08.08	84.83 ± 09.72	92.01 ± 06.50
PARIS	74	100.00 ± 00.00	98.65 ± 02.35	77.03 ± 40.00	88.51 ± 20.00	89.12 ± 07.18	91.94 ± 08.33	87.26 ± 08.29	87.93 ± 12.35	92.05 ± 09.16	86.36 ± 12.41
SLZ	49	100.00 ± 00.00	100.00 ± 00.00	100.00 ± 00.00	100.00 ± 00.00	89.28 ± 07.68	90.66 ± 07.50	88.75 ± 10.48	86.28 ± 10.65	87.45 ± 10.54	88.01 ± 10.66
STO	1283	99.45 ± 00.40	100.00 ± 00.00	100.00 ± 00.00	99.73 ± 00.20	88.06 ± 16.72	89.79 ± 15.47	88.99 ± 16.98	88.09 ± 16.12	89.22 ± 15.51	90.30 ± 14.47
STOP	442	97.29 ± 01.21	100.00 ± 00.00	100.00 ± 00.00	98.64 ± 00.60	84.03 ± 20.87	84.54 ± 22.02	85.30 ± 20.55	84.21 ± 20.63	84.49 ± 21.76	88.97 ± 13.93
SUH	251	99.60 ± 00.74	100.00 ± 00.00	100.00 ± 00.00	99.80 ± 00.37	88.43 ± 12.60	87.33 ± 14.45	91.11 ± 11.66	87.65 ± 13.23	86.37 ± 15.19	91.48 ± 10.17
UCSF	134	100.00 ± 00.00	99.25 ± 02.00	85.07 ± 40.00	92.54 ± 20.00	89.59 ± 07.91	89.50 ± 10.06	90.75 ± 08.01	88.72 ± 07.86	88.80 ± 09.89	90.18 ± 08.45
UMCG	86	100.00 ± 00.00	100.00 ± 00.00	100.00 ± 00.00	100.00 ± 00.00	88.61 ± 09.90	92.65 ± 10.94	86.53 ± 11.04	87.48 ± 10.49	91.18 ± 12.59	86.27 ± 10.15
UMCU	171	99.42 ± 01.03	100.00 ± 00.00	100.00 ± 00.00	99.71 ± 00.51	87.17 ± 09.55	90.60 ± 09.59	85.07 ± 12.07	86.11 ± 09.81	89.64 ± 10.57	84.85 ± 10.51
VUmc	97	100.00 ± 00.00	100.00 ± 00.00	100.00 ± 00.00	100.00 ± 00.00	89.81 ± 09.32	91.40 ± 08.86	89.58 ± 12.29	88.32 ± 10.22	89.76 ± 10.16	89.08 ± 12.50

There is a relationship between model performance and tumor size, the smaller the structure to detect the harder the task. The relationship is further outlined by the equally-populated boxplots showing the relation between tumor volume and voxel-wise Dice score (cf. left-hand side illustration in Fig. S1). The average voxel-wise Dice score is above 90% for tumors bigger than 6 ml, above 80% for tumors larger than 1.5 ml, and finally at 72% for tumor smaller than 1.5 ml. A total of 94 cases were completely missed, meaning a voxel-wise Dice score of 0%, of which 49 exhibit a tumor core smaller than 1 ml. The confidence interval

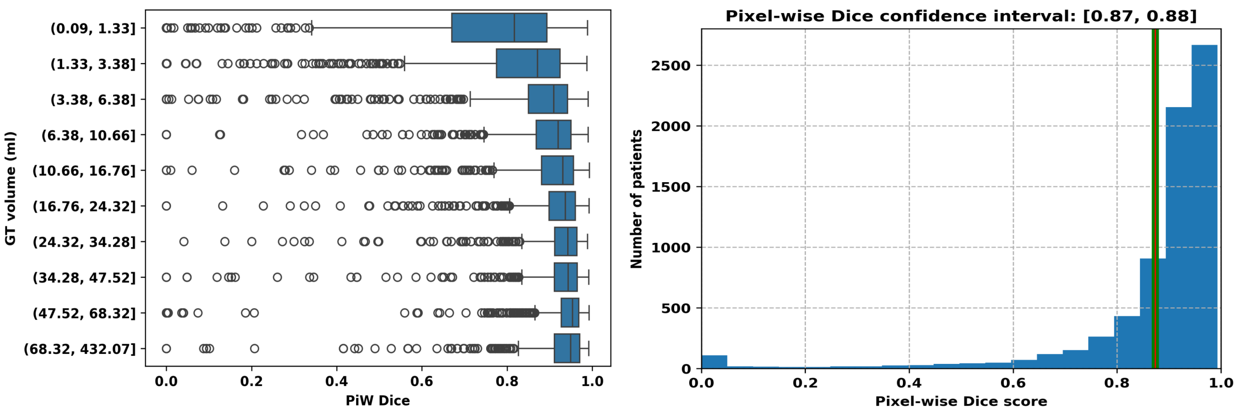

Figure S1. Boxplot showing the voxel-wise Dice against tumor core volume for ten equally populated bins (to the left) and voxel-wise confidence intervals (to the right) for all preoperative contrast-enhancing tumor core positive samples.

is closely around the reported average voxel-wise Dice score of 87.3%, indicating a low variability and high confidence in the estimated average (cf. right-hand side illustration in Fig. S1).

Regarding the NETC structure, a detailed performance summary is presented in Table. S8, for the segmentation model using t1c, t1w, and t2f MR sequences as inputs. Unsurprisingly, the model performs best on the glioma tumor type, where the non-enhancing tumor core structure is typically present with a relatively high volume. On the contrary, the model struggles in meningiomas as the NETC structure is rare and small (cf. Table S2), reaching only 56% Dice score. For equally small NETC structures, the Dice score is 12% higher over the metastasis group compared to the meningioma one. The positive rate in the former is 20% higher than in the latter, hence providing more positive sample during training.

In order to further investigate the model capabilities, a detailed summary is presented in Table. S9 illustrating the relationship between segmentation performances and stage of care. The highest Dice score, above 70%, was obtained over patient data acquired before surgery, where the tumor is potentially at its largest hence containing the most NETC. On the other hand, Dice scores over patient data acquired after surgery (i.e., early post-op and consecutive post-operative checks) are barely nearing 52%. Since, hopefully, the largest part of the tumor core has been removed during surgery, very little NETC is left to segment, making the task more difficult. In addition, potential confusion may arise between the post-operative NETC structure and resection cavity, both being visually very similar.

The average voxel-wise Dice score is above 80% for NETC bigger than 10 ml, around 75% for NETC larger than 3 ml, and finally at 50% for NETC smaller than 1 ml (cf. left-hand side illustration in Fig. S2). A total of 137 cases were completely missed, of which 108 exhibit a NETC structure smaller than 1 ml. The confidence interval is closely around the reported average voxel-wise Dice score of 66.7% (cf. right-hand side illustration in Fig. S2).

Table S8. NETC segmentation performance, per tumor type featured in the BraTS cohort, for the model using t1c, t1w, and t2f as inputs.

		Patient-wise				Voxel-wise			Object-wise		
Type	# Samples	Recall	Precision	Specificity	bAcc	Dice	Recall	Precision	Dice	Recall	Precision
GBM	2610	97.56 ± 01.59	85.85 ± 03.51	68.83 ± 08.48	83.19 ± 03.62	73.06 ± 26.18	79.18 ± 27.21	75.77 ± 24.08	73.64 ± 26.14	81.45 ± 26.95	76.17 ± 24.11
Meningioma	1000	76.27 ± 02.21	62.92 ± 02.55	76.50 ± 02.53	76.38 ± 00.68	56.14 ± 27.58	60.84 ± 31.91	63.12 ± 26.71	56.18 ± 32.90	71.46 ± 35.38	69.81 ± 29.15
Metastasis	817	96.62 ± 01.32	78.09 ± 06.63	67.29 ± 08.33	81.95 ± 04.21	69.79 ± 21.32	74.34 ± 25.35	72.56 ± 20.25	68.09 ± 26.11	77.47 ± 25.59	75.15 ± 21.52

Table S9. NETC segmentation performances per stage of care, for the model using t1c, t1w, and t2f as inputs.

		Patient-wise				Voxel-wise			Object-wise		
Fold	# Samples	Recall	Precision	Specificity	bAcc	Dice	Recall	Precision	Dice	Recall	Precision
Pre-op	2459	94.61 ± 01.36	87.51 ± 00.78	71.24 ± 02.78	82.93 ± 01.02	70.50 ± 29.75	74.92 ± 31.85	72.69 ± 28.20	70.02 ± 30.84	79.71 ± 30.36	77.49 ± 24.57
Follow-up	652	96.54 ± 01.71	77.74 ± 08.49	69.62 ± 09.41	83.08 ± 04.82	67.03 ± 24.86	71.19 ± 28.96	70.49 ± 24.15	65.17 ± 29.10	74.86 ± 28.94	74.94 ± 23.48
Early post-op	613	93.46 ± 05.93	64.09 ± 06.79	68.97 ± 09.25	81.21 ± 02.00	52.91 ± 31.23	62.94 ± 34.41	59.65 ± 31.43	55.86 ± 33.18	65.50 ± 36.14	67.77 ± 31.02
Post-op 1	484	97.40 ± 02.81	71.86 ± 05.68	76.26 ± 06.58	86.83 ± 02.84	54.11 ± 29.53	63.24 ± 33.35	60.32 ± 29.67	57.94 ± 31.33	68.75 ± 34.52	65.67 ± 30.21
Post-op 2	109	98.17 ± 04.00	69.58 ± 14.47	66.93 ± 12.36	82.55 ± 06.26	51.72 ± 30.17	65.85 ± 35.05	58.12 ± 30.20	59.60 ± 29.01	72.44 ± 33.65	66.58 ± 27.21
Post-op 3	70	100.00 ± 00.00	61.87 ± 22.68	72.84 ± 19.07	86.42 ± 09.54	52.90 ± 31.66	58.89 ± 35.98	66.92 ± 26.98	59.92 ± 25.07	66.08 ± 27.69	68.80 ± 29.10
Post-op 4	40	88.75 ± 20.00	80.33 ± 27.45	78.00 ± 32.00	83.37 ± 15.36	51.07 ± 27.71	59.41 ± 32.17	56.43 ± 30.41	51.89 ± 27.09	63.52 ± 32.06	61.98 ± 25.58

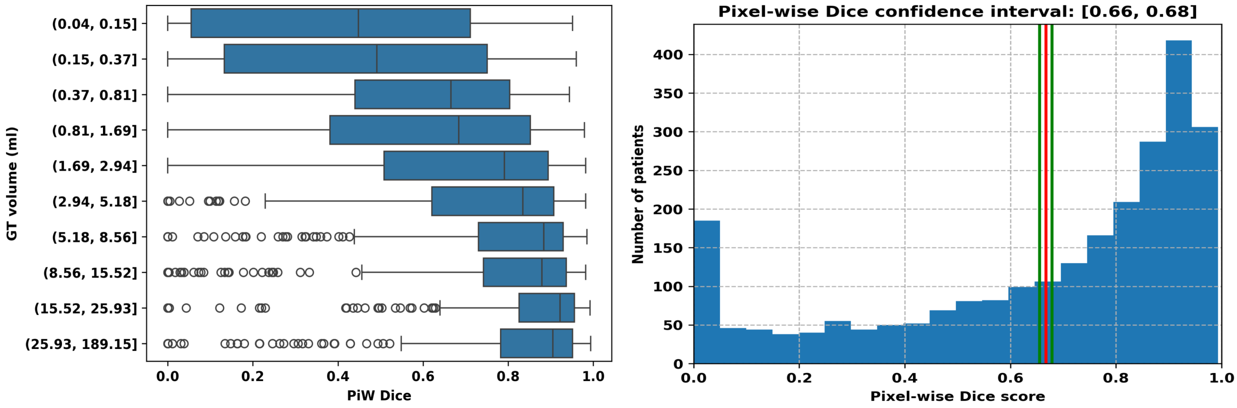

Figure S2. Boxplot showing the voxel-wise Dice against NETC volume for ten equally populated bins (to the left) and voxel-wise confidence intervals (to the right) for all positive NETC samples.

Table S10. Overall segmentation performance summary for postoperative contrast-enhancing residual tumor, using all four input sequences from dataset C.

		Patient-wise				Voxel-wise			Object-wise		
Fold	# Samples	Recall	Precision	Specificity	bAcc	Dice	Recall	Precision	Dice	Recall	Precision
HMC	40	93.00 ± 16.00	75.15 ± 05.05	37.08 ± 29.33	65.04 ± 14.85	29.24 ± 20.74	49.14 ± 32.22	33.53 ± 25.70	35.68 ± 22.84	56.21 ± 33.51	44.74 ± 30.93
HUM	5	60.00 ± 47.14	60.00 ± 47.14	80.00 ± 23.57	70.00 ± 35.36	19.07 ± 21.35	39.26 ± 41.66	18.46 ± 19.65	19.08 ± 21.35	39.26 ± 41.66	18.46 ± 19.65
BraTS	1316	97.48 ± 00.88	84.71 ± 02.89	59.63 ± 05.73	78.56 ± 02.80	77.45 ± 23.30	79.42 ± 22.86	79.42 ± 25.18	76.44 ± 25.27	82.41 ± 21.03	82.38 ± 22.18
MUW	44	97.73 ± 06.67	77.23 ± 20.84	56.82 ± 29.77	77.27 ± 13.15	51.81 ± 22.55	58.58 ± 26.03	53.92 ± 23.97	49.58 ± 17.99	59.60 ± 23.56	55.30 ± 20.77
PARIS	38	100.00 ± 00.00	75.66 ± 15.33	27.63 ± 40.00	63.82 ± 20.00	49.15 ± 17.48	64.75 ± 23.30	46.53 ± 19.05	49.00 ± 20.09	70.70 ± 25.01	46.02 ± 20.54
STO	407	95.89 ± 00.85	66.88 ± 03.84	45.12 ± 10.30	70.51 ± 05.51	59.83 ± 24.52	63.76 ± 28.19	66.00 ± 26.35	60.20 ± 24.58	67.48 ± 27.83	72.11 ± 22.85
SUH	199	75.13 ± 05.81	89.69 ± 08.84	83.83 ± 13.46	79.48 ± 05.97	44.01 ± 31.74	47.85 ± 35.45	47.55 ± 34.46	46.23 ± 32.13	51.22 ± 36.24	72.80 ± 26.50
UCSF	6	58.33 ± 41.46	58.33 ± 41.46	50.00 ± 43.30	54.17 ± 32.48	23.74 ± 36.93	27.10 ± 42.04	21.15 ± 33.01	24.61 ± 38.16	27.10 ± 42.04	47.54 ± 40.23
UMCG	43	97.67 ± 08.00	74.78 ± 13.71	32.44 ± 34.41	65.06 ± 13.78	43.86 ± 27.11	58.92 ± 33.02	43.36 ± 25.75	45.50 ± 26.87	65.05 ± 31.98	50.08 ± 27.82
UMCU	46	95.96 ± 05.71	62.73 ± 08.33	52.03 ± 12.00	74.00 ± 04.92	41.34 ± 21.97	66.30 ± 28.15	37.47 ± 24.29	42.13 ± 24.06	69.69 ± 29.47	47.45 ± 28.59
VUmc	72	96.30 ± 08.89	83.28 ± 05.58	29.68 ± 23.13	62.99 ± 09.00	49.07 ± 27.38	59.66 ± 31.65	50.15 ± 29.39	51.72 ± 28.61	64.08 ± 31.86	58.06 ± 30.59

2.2 Postoperative segmentation performances

A detailed cohort-wise performances summary is presented in Table. S10 for the postoperative contrast-enhancing residual tumor segmentation, using all four MR sequences as input. The cohorts not featuring any patient with all four sequences available were dropped from the table. The best voxel-wise Dice score was obtained for the BraTS cohort with 76%, followed by the STO cohort at 60%, and then most of the other cohorts at 45%. From the very limited number of patients with all MR sequences in many cohorts (i.e., less than 15 patients), it is difficult to judge whether the model struggled to generalize over out-of-distribution cases or not. On average, the residual tumor volumes are much larger over the BraTS cohort (15 ml), larger over the STO cohort (6 ml), and then around 4 ml for the other cohorts. The trend is clearly visible here also whereby a model struggles to segment smaller structures.

The trend is outlined by the equally-populated boxplots showing the relation between structure volume and voxel-wise Dice score (cf. left-hand side illustration in Fig. S3). An average voxel-wise Dice score of 80% was obtained for residual tumor bigger than 2 ml, 61% for residual tumor larger than 1.0 ml, and finally at 48% for residual tumor smaller than 1 ml. Out of the 1 375 positive cases with all four inputs available, 61 were completely missed by the model. The confidence interval is closely around the reported average voxel-wise Dice score of 70.2% (cf. right-hand side illustration in Fig. S3)

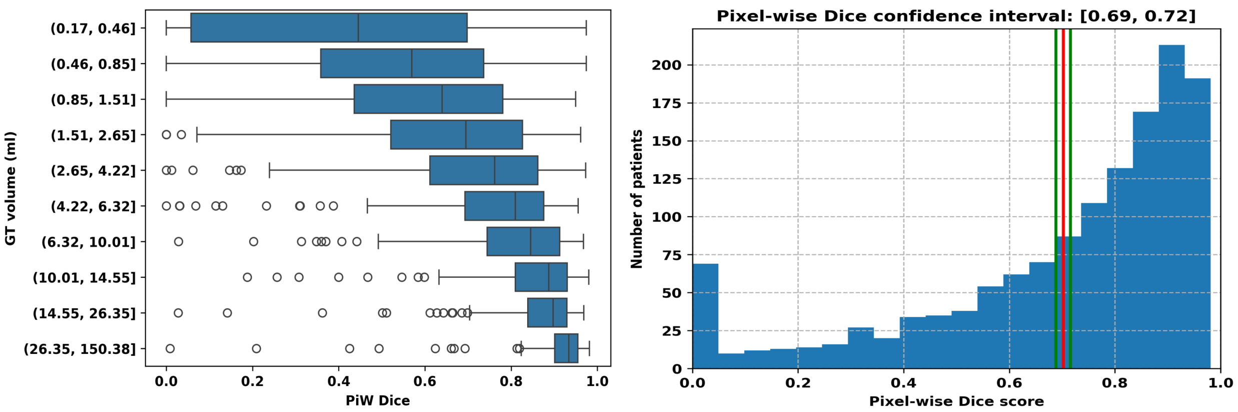

Figure S3. Boxplot showing the voxel-wise Dice against residual tumor volume for ten equally populated bins (to the left) and voxel-wise confidence intervals (to the right) for all positive residual tumor samples.

Finally, regarding the resection cavity structure, a detailed cohort-wise performances summary is presented in Table. S11, for the model using all four MR sequences as input. Across the board, the object-wise Dice scores are relatively stable, ranging from 77% (SUH cohort) up to 94% (PARIS cohort). For the two cohorts featuring some cases without any visible resection cavity, the patient-wise specificity and balanced accuracy are negatively impacted as the model has been trained with a bias towards expecting to segment a cavity.

Table S11. Resection cavity segmentation performances cohort-wise, using all four MR sequences as input.

		Patient-wise				Voxel-wise			Object-wise		
Type	# Samples	Recall	Precision	Specificity	bAcc	Dice	Recall	Precision	Dice	Recall	Precision
HMC	8	100.00 ± 00.00	100.00 ± 00.00	100.00 ± 00.00	100.00 ± 00.00	77.12 ± 26.19	75.76 ± 25.99	80.38 ± 29.82	77.12 ± 26.19	75.76 ± 25.99	80.38 ± 29.82
ISALA	5	100.00 ± 00.00	100.00 ± 00.00	100.00 ± 00.00	100.00 ± 00.00	88.16 ± 07.11	93.17 ± 04.86	84.82 ± 12.62	89.89 ± 04.15	93.17 ± 04.86	87.42 ± 07.66
BraTS	1316	99.55 ± 00.29	86.53 ± 02.06	19.58 ± 06.53	59.57 ± 03.16	75.18 ± 25.78	79.03 ± 25.22	77.00 ± 26.50	76.33 ± 25.49	79.78 ± 25.40	79.98 ± 25.01
MUW	14	100.00 ± 00.00	100.00 ± 00.00	100.00 ± 00.00	100.00 ± 00.00	87.87 ± 10.18	87.07 ± 12.98	91.48 ± 10.16	89.83 ± 08.98	87.07 ± 12.98	94.95 ± 04.81
PARIS	9	100.00 ± 00.00	100.00 ± 00.00	100.00 ± 00.00	100.00 ± 00.00	93.99 ± 00.94	93.15 ± 02.23	94.95 ± 02.38	94.01 ± 00.96	93.15 ± 02.23	94.98 ± 02.37
STO	275	99.64 ± 00.85	100.00 ± 00.00	100.00 ± 00.00	99.82 ± 00.43	81.63 ± 17.51	84.71 ± 17.94	81.65 ± 18.77	83.59 ± 16.66	84.53 ± 18.16	85.59 ± 15.74
SUH	165	100.00 ± 00.00	98.18 ± 02.69	58.18 ± 48.99	79.09 ± 24.49	76.02 ± 22.89	81.34 ± 22.14	76.14 ± 23.77	77.97 ± 21.60	81.62 ± 22.28	79.25 ± 21.56
UCSF	2	100.00 ± 00.00	100.00 ± 00.00	100.00 ± 00.00	100.00 ± 00.00	91.73 ± 07.04	90.83 ± 06.03	92.66 ± 08.10	91.76 ± 07.01	90.83 ± 06.03	92.71 ± 08.02
UMCG	11	100.00 ± 00.00	100.00 ± 00.00	100.00 ± 00.00	100.00 ± 00.00	88.42 ± 09.18	87.38 ± 12.72	91.46 ± 07.01	88.73 ± 12.04	86.30 ± 15.86	93.80 ± 03.47
UMCU	6	100.00 ± 00.00	100.00 ± 00.00	100.00 ± 00.00	100.00 ± 00.00	82.61 ± 11.08	86.41 ± 18.21	83.59 ± 13.25	83.12 ± 10.93	86.55 ± 18.18	84.21 ± 12.69
VUmc	15	100.00 ± 00.00	100.00 ± 00.00	100.00 ± 00.00	100.00 ± 00.00	87.86 ± 12.07	88.10 ± 10.64	88.92 ± 14.47	90.62 ± 05.80	88.10 ± 10.64	94.59 ± 02.83

Specifically for the resection cavity segmentation model using the t2f as single input, a detailed cohort-wise performances summary is presented in Table. S12. Surprisingly, the object-wise Dice score obtained over the BraTS cohort is only of 58%, under the average score of 66.5%, uncharacteristic from the previous results obtained this cohort both for the resection cavity and other structures. Conversely, the object-wise Dice score reaches at least 70% for the other two main cohorts (i.e., BOS and STO). An explanation might come from the type of CNS tumor featured in the different cohorts. The BOS cohort features only non contrast-enhancing tumors, while the BraTS cohort features predominantly contrast-enhancing tumors.

A similar trend as outlined before can be witnessed in the boxplots showing the relation between structure volume and voxel-wise Dice score (cf. left-hand side illustration in Fig. S4). An average voxel-wise Dice score of 85% was obtained for resection cavities bigger than 5 ml, and 59% for resection cavities smaller

Table S12. Resection cavity segmentation performances cohort-wise, using t2f only as input.

		Patient-wise				Voxel-wise			Object-wise		
Fold	# Samples	Recall	Precision	Specificity	bAcc	Dice	Recall	Precision	Dice	Recall	Precision
BOS	236	99.15 ± 01.05	99.58 ± 00.91	81.36 ± 40.00	90.25 ± 19.79	69.97 ± 22.29	76.13 ± 25.15	68.45 ± 21.90	70.05 ± 22.95	75.77 ± 26.06	71.07 ± 21.28
BraTS	1316	99.18 ± 01.05	85.18 ± 02.79	07.93 ± 03.05	53.55 ± 01.41	56.76 ± 31.90	62.01 ± 33.27	59.04 ± 32.21	58.16 ± 32.02	61.99 ± 33.90	61.88 ± 33.02
STO	70	100.00 ± 00.00	100.00 ± 00.00	100.00 ± 00.00	100.00 ± 00.00	76.82 ± 16.34	83.95 ± 19.37	74.88 ± 16.55	79.43 ± 15.03	85.37 ± 17.88	77.52 ± 16.43
UCSF	4	100.00 ± 00.00	100.00 ± 00.00	100.00 ± 00.00	100.00 ± 00.00	62.27 ± 11.54	96.47 ± 00.58	47.02 ± 12.53	62.34 ± 11.47	96.47 ± 00.58	47.09 ± 12.46

than 5 ml. Out of all the positive cases having all four inputs available, 34 were completely missed by the model. The confidence interval is closely around the reported average voxel-wise Dice score of 77.2% (cf. right-hand side illustration in Fig. S4)

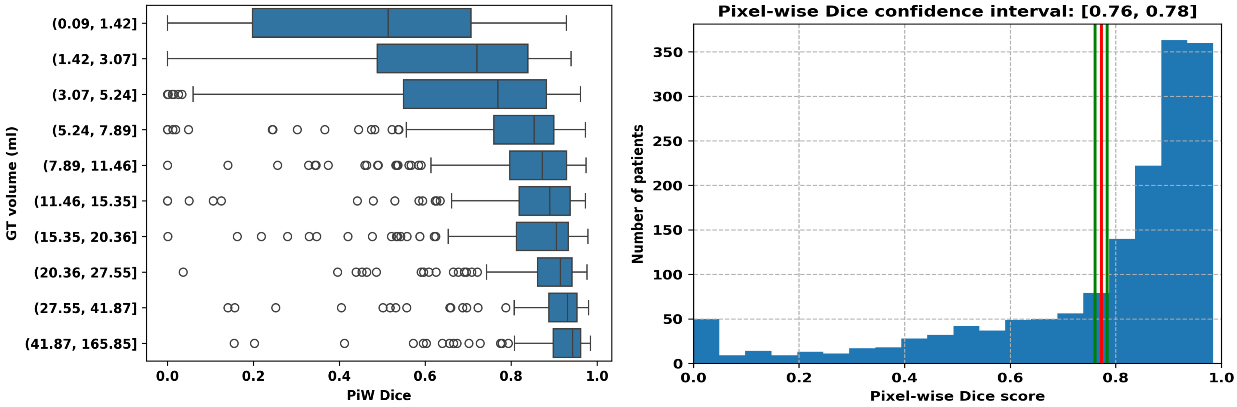

Figure S4. Boxplot showing the voxel-wise Dice against resection cavity volume for ten equally populated bins (to the left) and voxel-wise confidence intervals (to the right) for all positive resection cavity samples (with the model using all four MR scans as input).

2.3 Visual inspection of segmentation performance

An alternative version of Fig. 6, with higher magnification over the segmented regions, is provided in Fig. S5 to help better appreciate small differences between ground truth and model predictions.

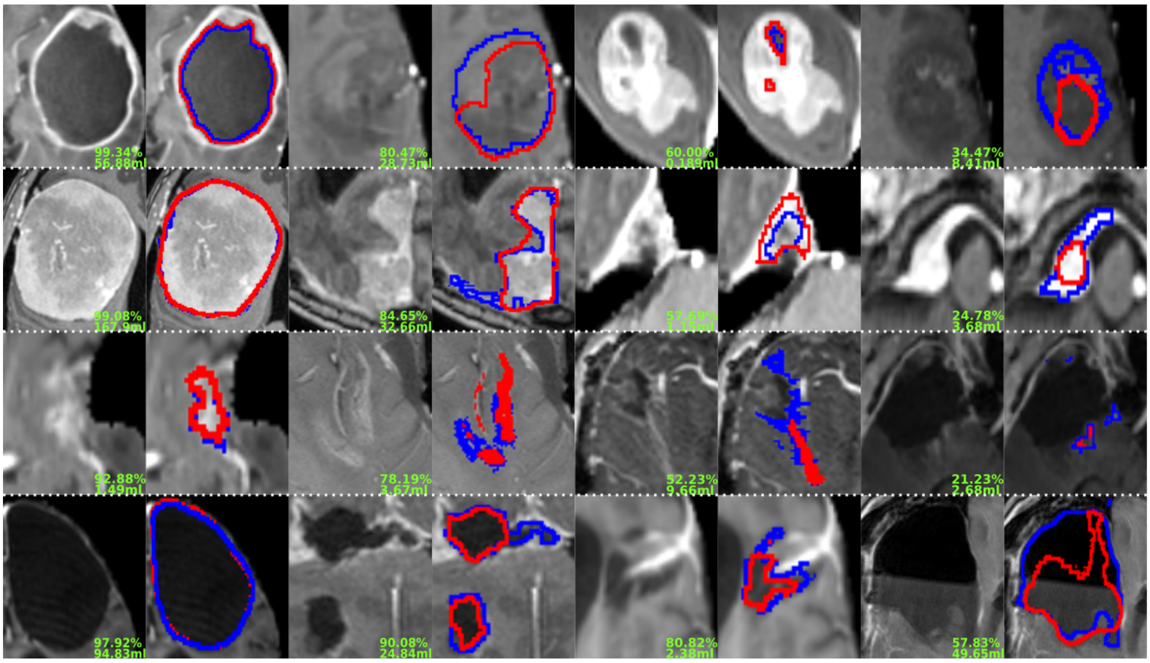

Figure S5. Zoomed in illustrations showing the ground truth (in blue) against the produced prediction (in red) for the NETC, tumor core, residual tumor, and resection cavity from to to bottom. The resulting pixel-wise Dice score and total volume to segment are given in green.

2.4 Generalizability investigation

2.4.1 Impact of acquisition conditions on model performance

For the STO cohort, we have grouped model performances for the TC and ET segmentation models according to different acquisitions. Tables S13- S16 report TC model performance against scanner manufacturer, scanner type, scanner field strength, and scanning sequence. Model performance is better on GE scanners that Philips or Siemens by 4%, however the sample size in the GE scanner category is relatively low. Performances are homogeneous across the major scanner types used, but a difference can be noted over volumes acquired with a 1T field strength (10+% Dice drop). Finally, and even if the sample size is limited, model performance seem to decrease considerably when the SE acquisition sequence is used as opposed to GR or GR/IR.

Table S13. Tumor core segmentation performances per scanner manufacturer, for the STO cohort and GBM tumor type.

		Patient-wise				Pixel-wise			Object-wise		
Manufacturer	# Samples	Recall	Precision	Specificity	bAcc	Dice	Recall	Precision	Dice	Recall	Precision
GE MEDICAL SYSTEMS	23	100.00 ± 00.00	100.00 ± 00.00	100.00 ± 00.00	100.00 ± 00.00	92.26 ± 04.99	92.64 ± 05.22	92.49 ± 07.50	91.68 ± 05.32	91.86 ± 06.17	92.58 ± 07.72
Philips Medical Systems	82	98.78 ± 01.67	100.00 ± 00.00	100.00 ± 00.00	99.39 ± 00.83	87.75 ± 17.52	88.06 ± 19.05	89.65 ± 16.11	87.29 ± 17.34	87.89 ± 18.54	90.40 ± 13.25
SIEMENS	487	99.18 ± 01.06	100.00 ± 00.00	100.00 ± 00.00	99.59 ± 00.53	87.65 ± 18.66	89.73 ± 16.56	88.81 ± 18.96	87.73 ± 18.35	89.21 ± 16.48	90.34 ± 16.59

Table S14. Tumor core segmentation performances per scanner type, for the STO cohort and GBM tumor type.

		Patient-wise				Pixel-wise			Object-wise		
Scanner type	# Samples	Recall	Precision	Specificity	bAcc	Dice	Recall	Precision	Dice	Recall	Precision
Achieva	12	100.00 ± 00.00	100.00 ± 00.00	100.00 ± 00.00	100.00 ± 00.00	90.38 ± 07.22	93.08 ± 06.68	89.25 ± 11.48	89.78 ± 07.03	92.39 ± 06.75	88.87 ± 11.73
Achieva dStream	1	100.00 ± 00.00	100.00 ± 00.00	100.00 ± 00.00	100.00 ± 00.00	97.29 ± 0	98.70 ± 0	95.91 ± 0	95.21 ± 0	96.84 ± 0	93.64 ± 0
Aera	4	100.00 ± 00.00	100.00 ± 00.00	100.00 ± 00.00	100.00 ± 00.00	94.91 ± 03.03	97.06 ± 02.91	93.13 ± 06.52	95.30 ± 02.35	97.06 ± 02.91	93.81 ± 05.25
Avanto	210	100.00 ± 00.00	100.00 ± 00.00	100.00 ± 00.00	100.00 ± 00.00	87.55 ± 17.03	90.21 ± 15.14	87.92 ± 18.16	88.00 ± 15.72	89.66 ± 14.48	89.29 ± 16.36
Avanto fit	20	100.00 ± 00.00	100.00 ± 00.00	100.00 ± 00.00	100.00 ± 00.00	90.60 ± 14.49	90.10 ± 17.21	93.43 ± 06.00	90.09 ± 14.36	89.91 ± 17.17	93.45 ± 05.24
Biograph mMR	17	100.00 ± 00.00	100.00 ± 00.00	100.00 ± 00.00	100.00 ± 00.00	95.56 ± 01.70	95.33 ± 03.86	95.99 ± 02.49	95.64 ± 01.68	95.33 ± 03.86	96.15 ± 02.40
GENESIS SIGNA	2	100.00 ± 00.00	100.00 ± 00.00	100.00 ± 00.00	100.00 ± 00.00	90.53 ± 04.38	89.12 ± 09.40	92.32 ± 01.03	89.42 ± 02.81	89.81 ± 10.38	89.94 ± 04.39
Ingenia	4	100.00 ± 00.00	100.00 ± 00.00	100.00 ± 00.00	100.00 ± 00.00	71.69 ± 47.42	70.86 ± 47.23	73.25 ± 47.15	67.36 ± 45.83	72.13 ± 48.14	65.53 ± 45.15
Ingenia Evolution	1	100.00 ± 00.00	100.00 ± 00.00	100.00 ± 00.00	100.00 ± 00.00	94.79 ± 0	97.30 ± 0	92.41 ± 0	94.79 ± 0	97.30 ± 0	92.41 ± 0
Intera	64	98.44 ± 02.11	100.00 ± 00.00	100.00 ± 00.00	99.22 ± 01.05	88.01 ± 16.22	87.88 ± 18.18	90.61 ± 13.76	87.83 ± 15.97	87.75 ± 17.51	92.16 ± 08.21
MAGNETOM Sola	5	100.00 ± 00.00	100.00 ± 00.00	100.00 ± 00.00	100.00 ± 00.00	89.02 ± 10.37	87.78 ± 17.33	92.92 ± 05.21	89.02 ± 10.37	87.78 ± 17.33	92.92 ± 05.21
OsiriX	1	100.0 ± 00.00	100.0 ± 00.00	100.0 ± 00.00	100.0 ± 00.00	96.34 ± 0	95.10 ± 0	97.62 ± 0	96.34 ± 0	95.10 ± 0	97.62 ± 0
Prisma	77	97.40 ± 03.81	100.00 ± 00.00	100.00 ± 00.00	98.70 ± 01.90	88.66 ± 18.35	87.80 ± 19.27	90.22 ± 18.75	88.38 ± 18.31	88.32 ± 16.90	93.32 ± 11.68
SIGNA Architect	2	100.00 ± 00.00	100.00 ± 00.00	100.00 ± 00.00	100.00 ± 00.00	89.48 ± 00.51	89.28 ± 02.34	89.76 ± 03.39	89.48 ± 00.51	89.28 ± 02.34	89.76 ± 03.39
SIGNA HDx	5	100.00 ± 00.00	100.00 ± 00.00	100.00 ± 00.00	100.00 ± 00.00	86.67 ± 06.08	89.19 ± 05.53	86.23 ± 12.40	85.79 ± 05.78	87.86 ± 06.07	87.02 ± 12.94
Signa HDxt	14	100.00 ± 00.00	100.00 ± 00.00	100.00 ± 00.00	100.00 ± 00.00	94.90 ± 02.54	94.85 ± 04.25	95.15 ± 03.13	94.41 ± 03.43	93.95 ± 05.73	95.35 ± 03.34
Skyra	125	98.40 ± 01.80	100.00 ± 00.00	100.00 ± 00.00	99.20 ± 00.90	85.60 ± 21.61	88.58 ± 18.53	87.80 ± 21.08	85.80 ± 21.72	88.70 ± 17.09	89.84 ± 18.53
Sonata	1	100.0 ± 00.00	100.0 ± 00.00	100.0 ± 00.00	100.0 ± 00.00	89.16 ± 0	91.32 ± 0	87.11 ± 0	88.36 ± 0	87.63 ± 0	91.57 ± 0
Symphony	10	100.00 ± 00.00	100.00 ± 00.00	100.00 ± 00.00	100.00 ± 00.00	89.65 ± 09.15	88.13 ± 09.91	93.18 ± 11.79	88.71 ± 10.30	86.62 ± 11.89	93.38 ± 11.06
TrioTim	17	100.00 ± 00.00	100.00 ± 00.00	100.00 ± 00.00	100.00 ± 00.00	84.15 ± 24.31	94.48 ± 03.09	82.96 ± 25.84	81.55 ± 26.63	84.41 ± 27.23	80.14 ± 27.17

Table S15. Tumor core segmentation performances per scanner field strength, for the STO cohort and GBM tumor type.

		Patient-wise				Pixel-wise			Object-wise		
Field strength	# Samples	Recall	Precision	Specificity	bAcc	Dice	Recall	Precision	Dice	Recall	Precision
1T	6	100.00 ± 00.00	100.00 ± 00.00	100.00 ± 00.00	100.00 ± 00.00	73.93 ± 31.11	70.22 ± 34.83	93.98 ± 05.92	74.47 ± 31.65	70.87 ± 34.87	94.91 ± 06.33
1.5T	292	100.00 ± 00.00	100.00 ± 00.00	100.00 ± 00.00	100.00 ± 00.00	88.22 ± 16.33	90.24 ± 15.28	88.84 ± 17.13	88.34 ± 15.34	89.69 ± 14.90	89.74 ± 15.76
3T	294	98.30 ± 02.01	100.00 ± 00.00	100.00 ± 00.00	99.15 ± 01.00	87.76 ± 19.49	89.39 ± 17.26	89.20 ± 19.50	87.58 ± 19.74	88.96 ± 17.35	91.04 ± 16.16

Table S16. Tumor core segmentation performances per scanning sequence, for the STO cohort and GBM tumor type.

		Patient-wise				Pixel-wise			Object-wise		
Sequence	# Samples	Recall	Precision	Specificity	bAcc	Dice	Recall	Precision	Dice	Recall	Precision
GR	90	98.89 ± 01.43	100.00 ± 00.00	100.00 ± 00.00	99.44 ± 00.71	90.69 ± 11.63	90.56 ± 13.31	91.77 ± 11.97	90.37 ± 11.35	90.14 ± 12.75	92.85 ± 07.38
GR/IR	468	99.13 ± 01.12	100.00 ± 00.00	100.00 ± 00.00	99.57 ± 00.56	87.50 ± 19.09	89.69 ± 16.83	88.63 ± 19.34	87.61 ± 18.76	89.22 ± 16.70	90.22 ± 16.90
SE	30	100.00 ± 00.00	100.00 ± 00.00	100.00 ± 00.00	100.00 ± 00.00	83.87 ± 20.96	85.70 ± 22.73	86.49 ± 18.16	82.59 ± 21.01	84.95 ± 23.08	85.76 ± 18.71
SE/IR	4	100.00 ± 00.00	100.00 ± 00.00	100.00 ± 00.00	100.00 ± 00.00	90.80 ± 03.27	90.33 ± 08.05	91.89 ± 04.02	89.88 ± 04.11	88.41 ± 09.88	92.98 ± 04.89

Tables S17- S20 report ET model performance against scanner manufacturer, scanner type, scanner field strength, and scanning sequence. The ET model used for this analysis was the model trained on all four MR sequences as input. Model performance is relatively similar between Philips and Siemens scanners. Larger performance variations across scanner types can be noticed, with a 30% decrease when using Avanto fit or Magnetom Sola, which could be due to their under-representation in the dataset. Based on three samples, model performance seem to decrease considerably when the SE acquisition sequence is used as opposed to GR or GR/IR. Overall, not enough samples are available in the under-represented categories to perform a robust multivariate analysis and draw any statistically significant conclusions. From visual inspection of the different tables, both TC and ET segmentation models seem robust and generalize well across scanner manufacturers, types, and scanning sequences.

Table S17. Enhancing residual tumor segmentation performances per scanner manufacturer for the STO cohort.

		Patient-wise				Pixel-wise			Object-wise		
Manufacturer	# Samples	Recall	Precision	Specificity	bAcc	Dice	Recall	Precision	Dice	Recall	Precision
Philips Medical Systems	33	100.00 ± 00.00	83.71 ± 04.64	21.21 ± 24.49	60.61 ± 12.25	62.35 ± 22.74	63.99 ± 23.66	68.91 ± 25.37	62.24 ± 22.72	72.01 ± 20.12	73.25 ± 21.64
SIEMENS	363	96.35 ± 02.18	65.39 ± 04.29	45.07 ± 11.00	70.71 ± 06.40	59.75 ± 24.18	63.97 ± 28.19	66.23 ± 25.76	60.17 ± 24.30	67.30 ± 28.02	71.56 ± 23.13

Table S18. Enhancing residual tumor segmentation performances per scanner type for the STO cohort.

		Patient-wise				Pixel-wise			Object-wise		
Scanner type	# Samples	Recall	Precision	Specificity	bAcc	Dice	Recall	Precision	Dice	Recall	Precision
Achieva	1	100.0 ± 00.00	100.0 ± 00.00	100.0 ± 00.00	100.00 ± 00.00	00.00 ± 0	00.00 ± 0	00.00 ± 0	00.00 ± 0	100.00 ± 0	100.00 ± 0
Avanto	177	96.72 ± 02.69	65.95 ± 09.72	31.45 ± 16.03	64.08 ± 08.26	60.27 ± 23.08	60.69 ± 28.16	70.35 ± 21.72	61.00 ± 22.66	64.09 ± 28.11	74.10 ± 19.65
Avanto fit	40	90.00 ± 40.00	31.17 ± 16.65	59.25 ± 09.80	74.62 ± 22.23	27.47 ± 24.63	57.40 ± 41.43	35.89 ± 40.39	33.16 ± 24.82	56.52 ± 41.86	41.05 ± 38.43
Intera	32	100.00 ± 00.00	83.15 ± 04.32	20.31 ± 24.49	60.16 ± 12.25	64.89 ± 19.62	66.60 ± 20.54	71.74 ± 21.99	64.80 ± 19.64	70.86 ± 19.73	72.20 ± 21.35
MAGNETOM Sola	8	50.00 ± 43.30	16.67 ± 14.43	54.17 ± 29.76	52.08 ± 14.88	33.62 ± 35.95	45.98 ± 49.16	26.50 ± 28.33	33.93 ± 36.27	46.21 ± 49.40	26.80 ± 28.65
Prisma	81	94.89 ± 07.00	66.95 ± 08.84	53.80 ± 18.34	74.34 ± 11.42	60.26 ± 25.99	68.00 ± 27.89	60.85 ± 29.12	59.14 ± 29.46	73.84 ± 28.86	67.44 ± 27.30
Skyra	50	100.00 ± 00.00	73.73 ± 21.20	53.21 ± 28.77	76.61 ± 14.38	63.77 ± 16.88	71.19 ± 20.01	66.10 ± 21.63	65.43 ± 14.75	70.35 ± 21.05	74.14 ± 17.61
TrioTim	15	86.67 ± 11.14	93.33 ± 20.00	86.67 ± 40.00	86.67 ± 18.47	57.81 ± 25.25	58.58 ± 28.77	61.75 ± 26.13	57.48 ± 25.00	66.90 ± 24.83	73.35 ± 18.52

Table S19. Enhancing residual tumor segmentation performances per scanner field strength for the STO cohort.

		Patient-wise				Pixel-wise			Object-wise		
Field strength	# Samples	Recall	Precision	Specificity	bAcc	Dice	Recall	Precision	Dice	Recall	Precision
1.5T	226	97.06 ± 02.40	61.01 ± 07.13	41.33 ± 09.47	69.19 ± 04.81	57.59 ± 24.99	60.64 ± 29.56	67.00 ± 25.56	58.79 ± 24.08	64.97 ± 28.90	72.16 ± 22.45
3T	178	96.36 ± 03.91	73.57 ± 06.72	51.04 ± 13.97	73.70 ± 07.96	62.63 ± 22.81	68.03 ± 25.03	65.37 ± 26.29	62.36 ± 24.11	71.64 ± 25.15	71.46 ± 23.24

Table S20. Enhancing residual tumor segmentation performances scanning sequence for the STO cohort.

		Patient-wise				Pixel-wise			Object-wise		
Sequence	# Samples	Recall	Precision	Specificity	bAcc	Dice	Recall	Precision	Dice	Recall	Precision
GR	53	96.23 ± 08.00	71.62 ± 07.57	40.39 ± 19.81	68.31 ± 10.31	60.11 ± 24.96	61.80 ± 26.86	67.46 ± 27.77	59.05 ± 26.44	69.31 ± 25.77	73.80 ± 24.27
GR/IR	348	97.30 ± 01.87	65.95 ± 04.52	45.73 ± 09.69	71.52 ± 05.68	60.51 ± 23.51	64.97 ± 27.53	66.73 ± 25.00	61.14 ± 23.28	67.43 ± 27.60	71.37 ± 22.60
SE	3	66.67 ± 50.00	33.33 ± 25.00	33.33 ± 50.00	50.00 ± 00.00	39.19 ± 33.94	41.09 ± 35.59	37.46 ± 32.44	38.45 ± 33.30	84.28 ± 13.61	67.78 ± 27.90

2.4.2 Sensitivity analysis: impact of detection threshold

The complete set of patient-wise, voxel-wise, and object-wise metrics are provided in Tables S21 and S22 to summarize model performance using varying detection thresholds. The voxel-wise and object-wise metrics are hence reported for the true positives only. In both, an increase of the detection threshold led to a decrease in recall while precision remained more stable. The decrease was larger for the ET segmentation model, which can be explained by the relationship between Dice as detection threshold and average structure volume. Small objects are more impacted in their Dice scores from slight under-/over-segmented compared to larger objects, which does not necessarily mean poor segmentation.

Table S21. Impact of the detection threshold on overall performances for the TC segmentation model.

		Patient-wise				Pixel-wise			Object-wise		
Threshold (%)	# Samples	Recall	Precision	Specificity	bAcc	Dice	Recall	Precision	Dice	Recall	Precision
0.1	7171	98.61 ± 00.27	99.73 ± 00.07	39.46 ± 16.84	69.04 ± 08.50	88.54 ± 13.33	88.66 ± 14.15	90.65 ± 12.29	88.06 ± 14.46	88.60 ± 14.14	90.97 ± 11.99
10	7171	98.19 ± 00.36	99.73 ± 00.07	39.46 ± 16.84	68.83 ± 08.54	88.90 ± 12.19	88.94 ± 13.33	90.83 ± 11.70	88.39 ± 13.45	88.93 ± 13.12	91.21 ± 11.03
25	7171	97.60 ± 00.46	99.73 ± 00.07	39.46 ± 16.84	68.53 ± 08.63	89.33 ± 10.87	89.24 ± 12.42	91.07 ± 10.92	88.78 ± 12.38	89.20 ± 12.23	91.38 ± 10.37
50	7171	95.69 ± 00.29	99.72 ± 00.07	39.46 ± 16.84	67.57 ± 08.53	90.32 ± 08.39	90.01 ± 10.47	91.73 ± 09.12	89.57 ± 10.57	89.77 ± 10.75	91.73 ± 09.34
75	7171	89.21 ± 00.43	99.70 ± 00.08	39.46 ± 16.84	64.34 ± 08.53	92.06 ± 05.23	91.73 ± 07.12	92.94 ± 06.62	91.17 ± 07.55	91.04 ± 08.44	92.64 ± 07.33

Table S22. Impact of the detection threshold on overall performances for the ET segmentation model, using all four sequences as input.

		Patient-wise				Pixel-wise			Object-wise		
Threshold (%)	# Samples	Recall	Precision	Specificity	bAcc	Dice	Recall	Precision	Dice	Recall	Precision
0.1	2224	95.54 ± 00.70	84.06 ± 02.56	70.61 ± 04.78	83.08 ± 02.48	73.52 ± 21.57	76.92 ± 21.85	76.48 ± 22.65	73.72 ± 21.59	78.20 ± 22.04	77.18 ± 22.60
10	2224	94.23 ± 00.82	83.89 ± 02.51	70.61 ± 04.78	82.42 ± 02.17	74.47 ± 20.16	77.78 ± 20.57	77.13 ± 21.78	74.55 ± 20.37	78.99 ± 20.84	77.80 ± 21.58
25	2224	91.51 ± 01.14	82.72 ± 02.63	68.92 ± 05.22	80.21 ± 02.35	76.10 ± 17.86	79.89 ± 18.63	77.41 ± 20.39	75.93 ± 18.46	81.21 ± 18.61	77.82 ± 20.40
50	2224	81.39 ± 01.36	81.83 ± 02.62	70.61 ± 04.78	76.00 ± 01.99	80.81 ± 12.51	82.54 ± 13.85	81.92 ± 15.90	80.39 ± 13.18	83.14 ± 14.40	81.80 ± 16.24
75	2224	57.56 ± 01.34	77.38 ± 03.87	72.63 ± 05.08	65.10 ± 02.75	87.65 ± 06.22	87.14 ± 08.20	89.09 ± 08.80	86.60 ± 08.45	86.97 ± 09.78	88.22 ± 10.41

2.4.3 Investigation of model struggles

A closer analysis of the results for the ET and NETC segmentation models was performed to identify reasons for large voxel-wise HD95 distances, negatively influencing the report standard deviations (cf. Fig.S6). For each patient, two different locations within the t1c MRI volumes are shown to properly illustrate the problems. In the first row, two regions were annotated and only one was predicted by the model. In the second row, only one region was annotated, close to the resection cavity. The other enhancing tumor component, not targeted during surgery, was not annotated but still segmented by the model. In the third row, the ground truth appears to be erroneous and the model did not make any prediction in that region. In the fourth row, the residual tumor close to the resection cavity was properly annotated but missed by the model. In addition, a second tumor component, not targeted during surgery, has been segmented fully (i.e., contrast-enhancing and necrosis core) while the model correctly segmented the enhancing tumor part. In the fifth row, both ground truth annotation and model predictions were correct regarding the NETC component. However, due to a noisy skull stripping process, the predictions also feature a wrong component outside of the actual brain region. In the sixth row, a similar side-effect to skull stripping is visible whereby in addition to a correct NETC prediction, a wrong and tiny one is featured at the edge of the brain. Finally in the last row, ground truth annotation noise is visible which is correctly not detected by the model and only the proper NETC components are segmented. Overall, three main problems arise from this visual inspection: (i) noise in the ground truth, (ii) annotation inconsistencies in the ground truth, and (iii) side-effects from processing skull-stripped volumes.

Visual inspection of hard cases for the ET and RC models, are provided in Figures S7 and S8. For the ET segmentation model, the first presents a case where no residual tumor has been annotated and where the model correctly did not predict residual tumor. However, another contrast-enhancing tumor component is also present in the brain, not targeted during surgery, and which has not been annotated. Since the model correctly segmented all contrast-enhancing regions for this patient, reported model performances are poor even though model predictions are correct. The second case is similar, but also contains false positive predictions from the model over the resected area. Finally, in the last case, the annotation performed on the postoperative MR scan covers another tumor component than the one annotated on the preoperative MR scan and targeted during surgery. For the RC segmentation model, the first case (top-left) features what seems to be a resection cavity, which was correctly segmented, but not annotated. Such case might be a re-operation, close to an old resection location, which did not further extend the resection cavity hence the lack of annotation. Access to preoperative imaging for this case would be needed for confirmation. In the second case (top-right), the NETC region, looking very similar to a RC, has been mistakenly segmented. This is a common pitfall for the model as it has not been exposed to NETC structures and lacks context

to understand which tumor component where surgically targeted and which were not. In the third case (middle row), a mix of RC and NETC was segmented by the model. Finally, in the last case (bottom row), a SNFH structure was mistaken for RC. All these cases highlight the struggles of the RC model to be specific to resection cavities, often segmenting similar looking structures (e.g., NETC), and the inconsistencies in annotation with respect to old resection cavities.

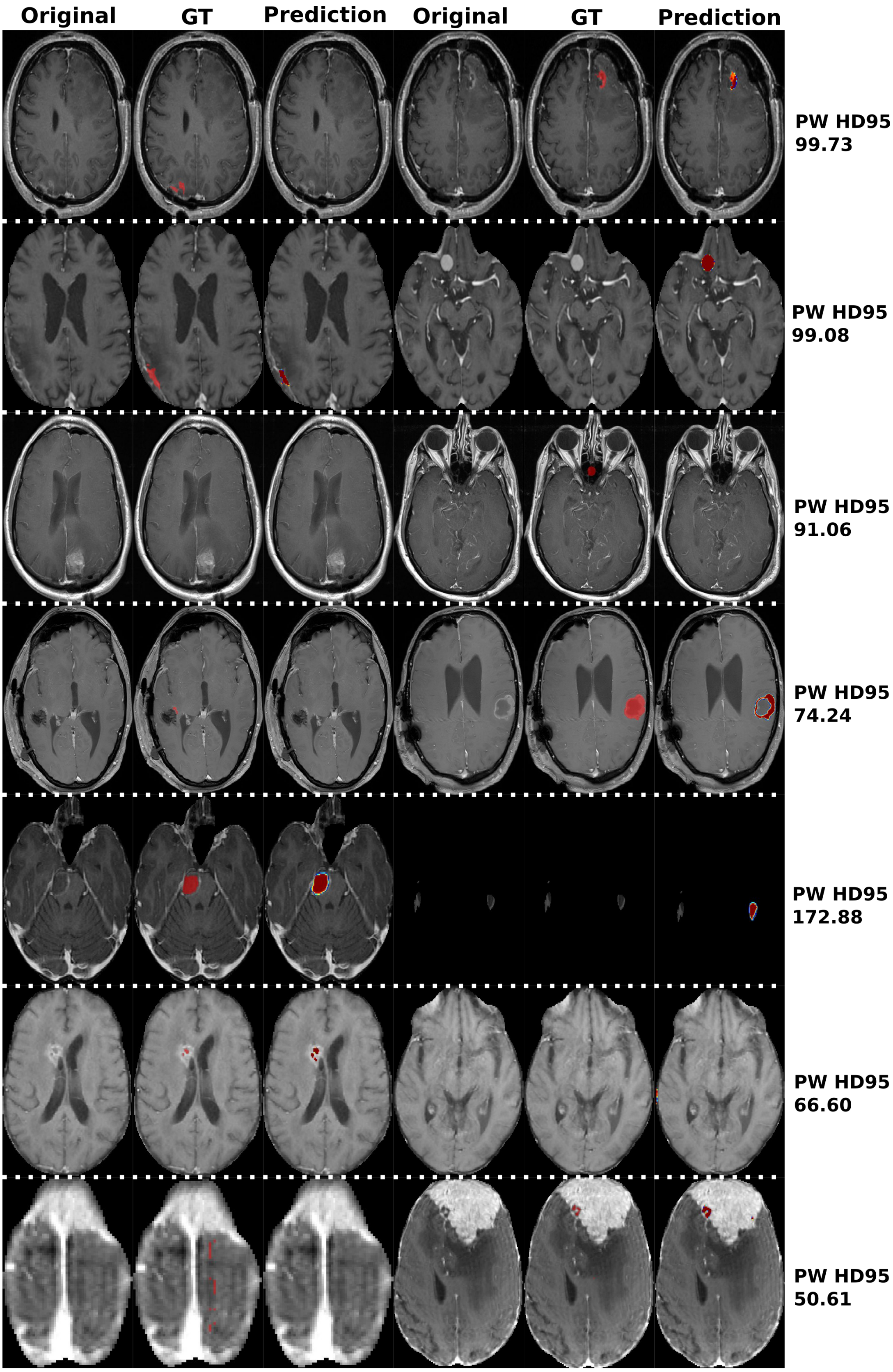

Figure S6. Illustrations of cases with high voxel-wise HD95 values, one per row. The first four rows feature predictions from the ET segmentation model and the last three rows feature predictions form the NETC segmentation model. The manual ground (GT) is shown in red, and the probabilistic model predictions are shown as overlaid heatmaps.

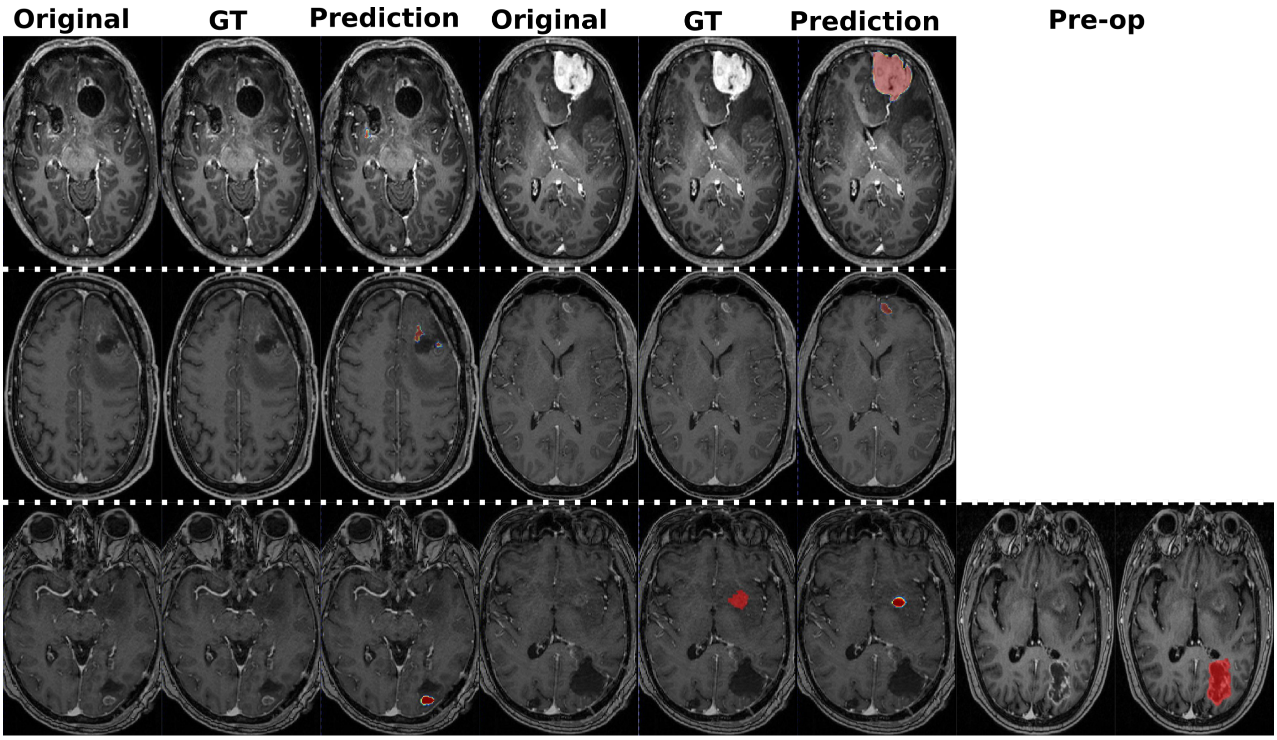

Figure S7. Hard cases for the ET segmentation task where each row represents a different case.

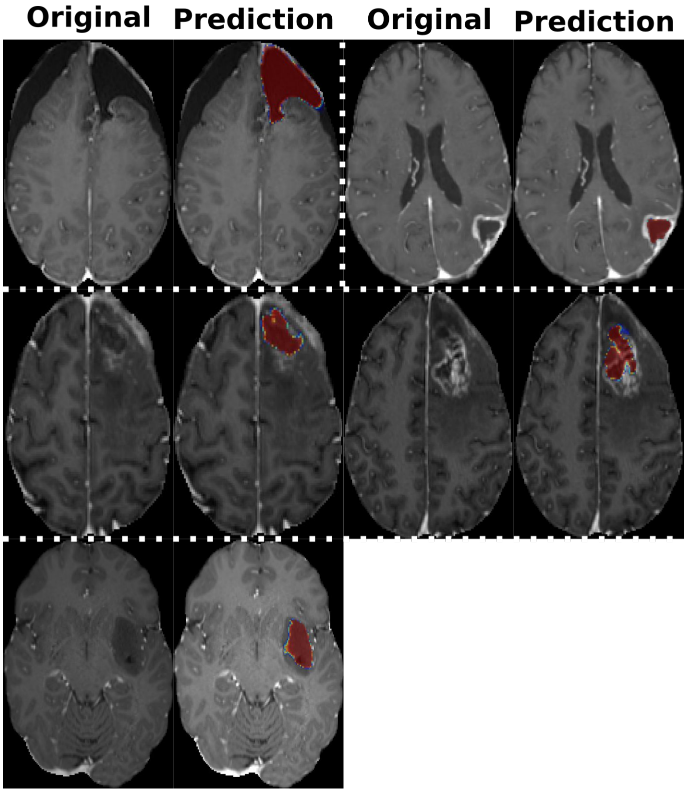

Figure S8. Hard cases for the RC segmentation task where the dotted white lines separate the different cases.
